# Complement receptor 3 mediates both sinking phagocytosis and phagocytic cup formation *via* distinct mechanisms

**DOI:** 10.1016/j.jbc.2021.100256

**Published:** 2021-01-08

**Authors:** Stefan Walbaum, Benjamin Ambrosy, Paula Schütz, Anne C. Bachg, Markus Horsthemke, Jeanette H.W. Leusen, Attila Mócsai, Peter J. Hanley

**Affiliations:** 1 Institut für Molekulare Zellbiologie, Westfälische Wilhems-Universität Münster, Münster, Germany; 2 Center for Translational Immunology, University Medical Center Utrecht, Utrecht, The Netherlands; 3 Department of Physiology, Semmelweis University School of Medicine, Budapest, Hungary; 4 Department of Physiology, Pathophysiology and Toxicology and ZBAF (Centre for Biomedical Education and Research), Faculty of Health, School of Medicine, Witten/Herdecke University, Witten, Germany

**Keywords:** Complement system, knockout mice, live-cell imaging, macrophages, phagocytosis, C5 null, C5-deficient, cKO, conditional KO, CMO, CellMask Orange, dKO, double KO, FCS, fetal calf serum, hRBCs, human red blood cells, IgG, immunoglobulin G, IgM, immunoglobulin M, ITAM, immunoreceptor tyrosine-based activation motif, ITIM, immunoreceptor tyrosine-based inhibitory motif, NOTAM, No ITAM

## Abstract

A long-standing hypothesis is that complement receptors (CRs), especially CR3, mediate sinking phagocytosis, but evidence is lacking. Alternatively, CRs have been reported to induce membrane ruffles or phagocytic cups, akin to those induced by Fcγ receptors (FcγRs), but the details of these events are unclear. Here we used real-time 3D imaging and KO mouse models to clarify how particles (human red blood cells) are internalized by resident peritoneal F4/80^+^ cells (macrophages) *via* CRs and/or FcγRs. We first show that FcγRs mediate highly efficient, rapid (2–3 min) phagocytic cup formation, which is completely abolished by deletion or mutation of the FcR γ chain or conditional deletion of the signal transducer Syk. FcγR-mediated phagocytic cups robustly arise from any point of cell-particle contact, including filopodia. In the absence of CR3, FcγR-mediated phagocytic cups exhibit delayed closure and become aberrantly elongated. Independent of FcγRs, CR3 mediates sporadic ingestion of complement-opsonized particles by rapid phagocytic cup-like structures, typically emanating from membrane ruffles and largely prevented by deletion of the immunoreceptor tyrosine-based activation motif (ITAM) adaptors FcR γ chain and DAP12 or Syk. Deletion of ITAM adaptors or Syk clearly revealed that there is a slow (10–25 min) sinking mode of phagocytosis *via* a restricted orifice. In summary, we show that (1) CR3 indeed mediates a slow sinking mode of phagocytosis, which is accentuated by deletion of ITAM adaptors or Syk, (2) CR3 induces phagocytic cup-like structures, driven by ITAM adaptors and Syk, and (3) CR3 is involved in forming and closing FcγR-mediated phagocytic cups.

Phagocytosis, receptor-mediated ingestion of particles larger than 0.5 μm in diameter, is the major effector function of macrophages (big eaters) and neutrophils, previously known as microphages (small eaters) (1). Surprisingly, more than 100 years after Metschnikoff developed his theory of phagocytosis (2), the mechanisms of ingestion are not well understood (3). In a pioneering study, Munthe-Kaas *et al.* (4) and Kaplan (5) described two distinctive modes of phagocytosis based on high-resolution snapshots obtained by scanning electron microscopy. Specifically, Kaplan deduced that mouse peritoneal macrophages engulfed immunoglobulin G (IgG)-opsonized sheep red blood cells “by means of thin membrane extensions rising from the macrophage surface and enclosing the opsonized particles tightly in a cup-like structure protruding from the macrophage surface” (5), in accord with the zipper model of phagocytosis (6). In contrast, complement-opsonized sheep red blood cells appeared to directly sink into macrophages without the involvement of membrane protrusions (4, 5). The two modes of phagocytosis, phagocytic cup formation and sinking phagocytosis, have become well established in the literature (7, 8, 9, 10, 11). However, at variance with the notion of two morphologically distinct modes, transmission electron micrographs revealed membrane extensions during both Fcγ receptor (FcγR)- and CR-mediated phagocytosis (12), and high-resolution surface imaging showed prominent local membrane ruffles around complement-opsonized sheep red blood cells attached to RAW264.7 cells (13), a macrophage cell line. Furthermore, time-lapse 2D confocal microscopy of RAW264.7 macrophages expressing fluorescently labeled actin indicated that thin, actin-rich membrane extensions envelop complement-opsonized sheep red blood cells (14). Similarly, Jaumouillé *et al*. (15) recently observed phagocytic cup formation during the phagocytosis of iC3b-coated polystyrene beads by RAW264.7 and bone marrow–derived Lifeact-EGFP macrophages. Thus, it remains to be established whether complement receptor–mediated phagocytosis involves sinking in, reaching up (of the membrane), or both.

Here we used RNA-sequence analysis, genetic experiments, and real-time 3D imaging to explore the roles of FcγRs and complement receptors in phagocytosis. We systematically analyzed how resident macrophages, isolated from WT or KO mice, engage and internalize variously opsonized human red blood cells (hRBCs) using time-lapse spinning disk confocal microscopy.

## Results

### Expression of FcγRs in mouse macrophages and real-time assay for complement-mediated hemolysis triggered by IgG-opsonized hRBCs

Using RNA-sequence analysis, we first determined the expression profile of FcγR and upstream signaling molecules in mouse resident peritoneal F4/80^+^ cells (macrophages), which had been purified by cell sorting (Fig. S1*A*). All four FcγRs (FcγRI, FcγRIIb, FcγRIII, and FcγRIV) and the Fc receptor γ chain (encoded by *Fcer1g*) could be detected, as well as transcripts encoding key signal transducers, the tyrosine kinase Syk (*Syk*), also known as spleen (S) tyrosine (Y) kinase, and the tyrosine phosphatases Shp1 (*Ptpn6*) and Shp2 (*Ptpn11*). Three of the four FcγRs (FcγRI, FcγRIII, and FcγRIV) bind noncovalently to the Fc receptor γ chain, which contains an immunoreceptor tyrosine-based activation motif (ITAM), whereas FcγRIIb contains an immunoreceptor tyrosine-based inhibitory motif (ITIM) in its cytosolic tail (Fig. S1*B*). Among the three FcγRs associated with the Fc receptor γ chain, FcγRIII (*Fcgr3*) was the most strongly expressed.

FcγR-mediated phagocytosis has commonly been investigated using IgG-opsonized sheep red blood cells. Here we used IgG-opsonized hRBCs, freshly isolated on the day of experiments. hRBCs were opsonized with monoclonal mouse anti-human CD235a (subclass IgG2b) antibodies, which should be recognized by all mouse FcγRs with moderate to high affinity (16). Incubation of IgG-opsonized hRBCs with mouse WT serum should trigger the classical complement cascade, leading to deposition of complement C3b and hemolysis upon formation of the membrane attack complex (Fig. S1*C*). We developed real-time hemolysis assays to confirm that opsonization with mouse IgG antibodies was sufficient to induce hemolysis, indexed as loss of cytosolic calcein fluorescence, whereas the red fluorescent probe CellMask Orange (CMO) served as a plasma membrane marker (Fig. S1, *D* and *E*). Indeed, the introduction of WT serum to IgG-opsonized hRBCs induced hemolysis, such that more than 95% of cells were lyzed between 3 and 6 min (Fig. S1*F*).

### Expression of CRs in mouse macrophages and real-time assay for complement-mediated hemolysis triggered by immunoglobulin M (IgM)-opsonized hRBCs

At least five CRs (CR1, CR2, CR3, CR4, and CRIg) are implicated in the binding of immune cells to complement C3b and its proteolytic fragment iC3b (Fig. S2*A*). RNA-sequence analysis indicated that CR3 and CRIg (encoded by *Vsig4*) are expressed in mouse peritoneal F4/80^+^ cells (macrophages) (Fig. S2*B*), whereas expression of CR1 and CR2 (both encoded by *Cr2*) and CR4 could not be detected, consistent with previous work (17). CR3 and CR4 are integrins, cell-extracellular matrix adhesion molecules, which share the same β_2_-chain (CD18), encoded by *Itgb2*, but have different α-chains. The α-chain of CR3 (also known as CD11b) is encoded by *Itgam*, whereas the α-chain of CR4 (also known as CD11c), a dendritic cell marker, is encoded by *Itgax*. CRIg binds C3b and iC3b (18, 19) and was reported to play an important role in complement-mediated clearance of pathogens from the circulation (18). Notably, isolated resident peritoneal cells predominantly consist of F4/80^+^ cells (macrophages) and CD19^+^ cells (B cells), although low levels (less than 5% of total population) of CD19^–^/CD3^+^ cells (T cells) and F4/80^–^/CD11c^+^ cells (dendritic cells) can be detected. In our phagocytosis assays, Alexa Fluor 488–conjugated anti-F4/80 antibodies served as both an unambiguous macrophage marker and a fluorescent membrane probe.

We developed assays to study complement C3b/iC3b-mediated phagocytosis using either IgG (Fig. S1*C*) or IgM (Fig. S2*C*) to trigger activation of the classical complement cascade. To test the efficacy of IgM opsonization, we loaded the cytosol of hRBCs with the pH-sensitive probe pHrodo Red using its cell-permeable acetoxymethyl (AM) ester form (Fig. S2*D*) and incubated either unopsonized or IgM-opsonized hRBCs with WT mouse serum (Fig. S2*E*). Loss of pHrodo Red fluorescence was used as a readout of hemolysis (Fig. S2*F*). Importantly, real-time hemolysis assays using pHrodo Red–loaded hRBCs were performed after opsonizing cells with IgM at room temperature (RT) and slowly increasing the temperature to ∼30 °C (Fig. S2*G*). This empirical protocol was a compromise taking into account that, on the one hand, IgM has weak affinity for antigens at 37 °C and, on the other hand, the activity of the complement cascade decreases at lower temperatures. More than 50% of IgM-opsonized hRBCs lyzed within 8 min after application of WT mouse serum (Fig. S2*G*). In summary, we measured the expression profile of phagocytic receptor–related genes in the cells used in this study (resident peritoneal F4/80^+^ cells), and we showed using real-time hemolysis assays that the IgG- and IgM-opsonization protocols robustly induced hemolysis upon application of WT serum.

### Imaging of FcγR-mediated phagocytic events

**Figure 1:**
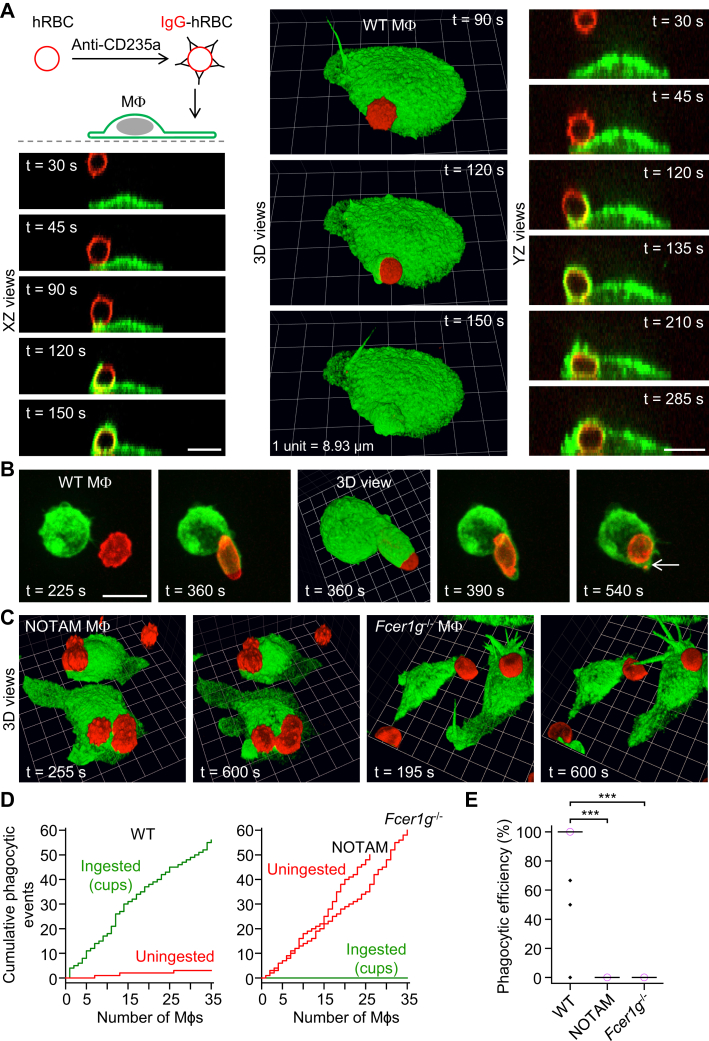
**Phagocytic cup formation mediated by Fcγ receptors.***A*, 3D time-lapse images obtained by spinning disk confocal microscopy showing a WT macrophage (Mϕ) ingesting a human red blood cell (hRBC) opsonized with mouse IgG antibodies (mouse anti-human CD235a monoclonal IgG2b antibodies). The macrophage was labeled green fluorescent with Alexa Fluor 488–conjugated anti-F4/80 antibodies, and hRBCs were labeled red fluorescent with the plasma membrane stain CellMask Orange. Scale bars, 10 μm. *B*, ingestion of an IgG-opsonized hRBC by a morphologically spherical macrophage. Note that the tip of the ingested and squeezed hRBC is pinched off during cup closure (*white arrow*). This may serve as an immune defense strategy to lyse ingested cells. Scale bar, 10 μm (grid spacing [3D view] = 3.05 μm). *C*, lack of ingestion of IgG-opsonized hRBCs by No ITAM (NOTAM) (grid spacing = 6.46 μm) and *Fcer1g*^−/−^ macrophages (grid spacing = 6.49 μm). NOTAM macrophages contain nonsignaling immunoreceptor tyrosine-based activation motifs (ITAMs) in the Fc receptor γ chain. *D*, cumulative plots of phagocytic (ingestion) events for WT, NOTAM, and *Fcer1g*^−/−^ macrophages. The plots for NOTAM and *Fcer1g*^−/−^ macrophages are overlaid. The *x*-axes show individual macrophages. *E*, summary box plots of phagocytic efficiency, defined as the percentage of IgG-opsonized hRBCs (in contact with a macrophage) which were fully ingested. Medians are represented by a *horizontal line* with a *lilac-colored circle*. Notably, following the introduction of IgG-opsonized hRBCs, the phagocytic efficiencies of NOTAM and *Fcer1g*^−/−^ macrophages were negligible, whereas WT macrophages were highly efficient eaters. Data were analyzed using the Kruskal–Wallis one-way ANOVA (H = 89.8, degrees of freedom = 2, and *p* < 0.0001). Post hoc comparisons were performed using the Dunn test; *n* (number of macrophages) = 35 (from 3 WT mice), *n* = 26 (from 4 NOTAM mice), and *n* = 35 (from 4 *Fcer1g*^−/−^ mice). ∗∗∗*p* < 0.0001. IgG, immunoglobulin G.

Control experiments confirmed that mouse peritoneal macrophages did not ingest unopsonized hRBCs, providing a means to study FcγR-mediated phagocytosis with negligible background phagocytic activity. hRBCs were opsonized with mouse monoclonal anti-human CD235a IgG antibodies and introduced to mouse macrophages labeled with green fluorescent (Alexa Fluor 488 conjugated) anti-F4/80 antibodies (Fig. 1*A*). Time-lapse spinning disk confocal microscopy was performed to visualize single FcγR-mediated phagocytic events (Fig. 1*A*; Videos S1 and S2). Macrophages ingested IgG-conjugated hRBCs *via* phagocytic cup formation, irrespective of whether the hRBC was engaged by a morphologically spread out (Fig. 1*A*; Video S1) or spherical macrophage (Fig. 1*B*; Video S2). The tip of hRBCs, engulfed and squeezed by phagocytic cups, was frequently pinched off, suggesting that forceful cup closure may serve to lyse cells (Fig. 1*B*). Phagocytic cup formation was not observed in macrophages isolated from No ITAM (NOTAM) mice (Fig. 1*C*, two frames on the left), which have a nonsignaling ITAM in the Fc receptor γ chain, or *Fccer1g*^−/−^ (Fc receptor γ chain KO) mice (Fig. 1*C*, two frames on the right). Sinking phagocytosis was not observed in either NOTAM or *Fcer1g*^−/−^ macrophages presented with IgG-opsonized hRBCs. Cumulative plots of phagocytic events (*y*-axis) by individual macrophages (*x*-axis) clearly show that WT macrophages efficiently ingest IgG-opsonized hRBCs by phagocytic cup formation and few targets remain uningested, whereas IgG-opsonized hRBCs in contact with NOTAM or *Fcer1g*^−/−^ macrophages are not ingested (Fig. 1*D*). In summary, these data show that the ingestion of IgG-opsonized hRBCs is mediated by phagocytic cup formation and requires the Fc receptor γ chain and a functional ITAM (Fig. 1, *D* and *E*), and sinking phagocytosis does not prevail in either *Fcer1g*^−/−^ or NOTAM macrophages. Interestingly, phagocytic cup closure may pinch off the tip, and presumably induce lysis, of ingested cells.

### NOTAM and Fcer1g^−/−^ macrophages ingest dual IgG- and C3b/iC3b-opsonized hRBCs to varying degrees

**Figure 2:**
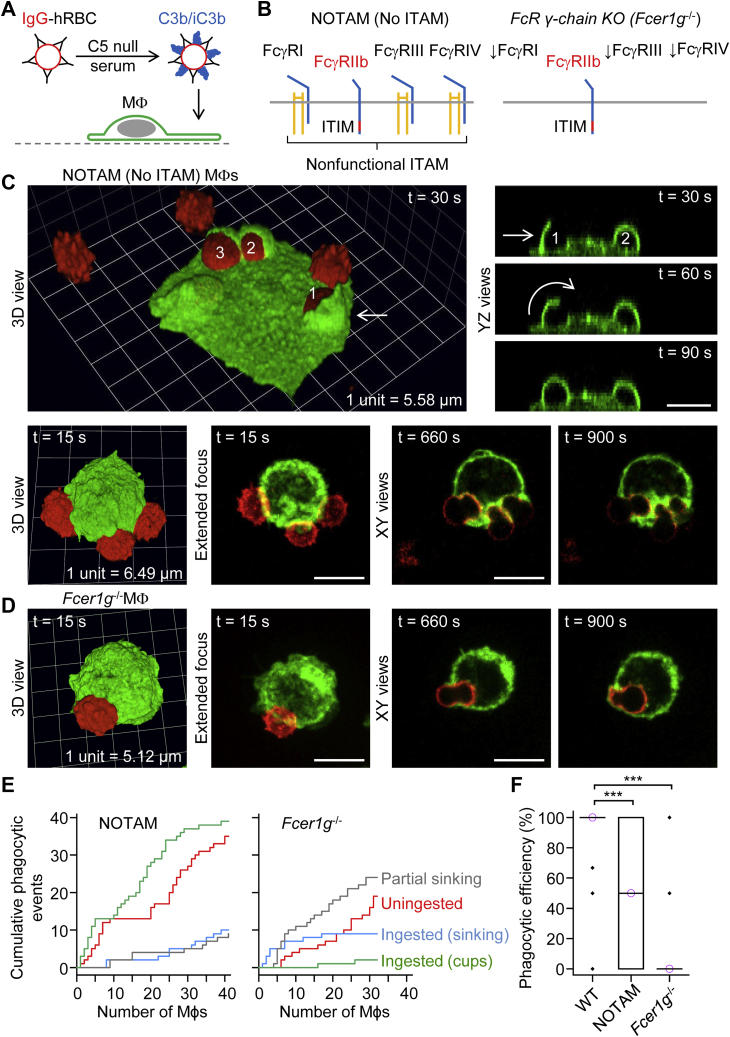
**Ingestion of dual IgG- and complement C3b/iC3b-opsonized human red blood cells by NOTAM and *Fcer1g***^**−/−**^**macrophages.***A*, human red blood cells (hRBCs) were dual-opsonized with IgG and complement C3b/iC3b by incubating IgG-opsonized hRBCs with complement C5-deficient (C5 null) mouse serum. *B*, macrophages isolated from No ITAM (NOTAM) mice contain nonsignaling immunoreceptor tyrosine-based activation motifs (ITAMs) in the Fc receptor γ chain, whereas the Fc receptor γ chain (encoded by *Fcer1g*) is deleted in macrophages from *Fcer1g*^−/−^ mice, leading to loss of expression of the α-chains FcγRI, FcγRIII, and FcγRIV. The immunoreceptor tyrosine-based inhibition motif (ITIM)-containing FcγR, FcγRIIb, is functional in both NOTAM and *Fcer1g*^−/−^ macrophages. *C*, ingestion of dual IgG- and C3b/iC3b-opsonized hRBCs by NOTAM macrophages *via* phagocytic cup formation (upper panel) or partial sinking phagocytosis (lower panel). The phagocytic cups in the upper panel arose from the extension of membrane protrusions, which rolled over the particle (hRBC). Particle number 3 was not ingested because of retraction of the membrane protrusion. 3D time-lapse imaging was performed for 16 min by spinning disk confocal microscopy. Scale bars, 10 μm. *D*, ingestion of a dual IgG–opsonized and C3b/iC3b-opsonized hRBC by a *Fcer1g*^−/−^ macrophage *via* sinking phagocytosis. Scale bar, 10 μm. *E*, cumulative plots of phagocytic (ingestion) events for NOTAM and *Fcer1g*^−/−^ macrophages. The *x*-axes show individual macrophages. *F*, summary box plots of macrophage phagocytic efficiency, defined as the percentage of IgG-opsonized hRBCs (in contact with a macrophage) which were fully ingested. Medians are represented by a *horizontal line* with a *lilac-colored circle*. Data were analyzed using the Kruskal–Wallis one-way ANOVA (H = 50.4, degrees of freedom = 2, and *p* < 0.0001). Post hoc comparisons were performed using the Dunn test; *n* (number of macrophages) = 35 (from 3 WT mice), *n* = 41 (from 4 NOTAM mice), and *n* = 31 (from 2 *Fcer1g*^−/−^ mice). ∗∗∗*p* < 0.0001. IgG, immunoglobulin G.

hRBCs were dual-opsonized with IgG and complement C3b/iC3b by incubating IgG–hRBCs with complement C5-deficient (C5 null) serum (Fig. 2*A*) and introduced to NOTAM or *Fcer1g*^−/−^ macrophages, the latter of which do not exhibit surface expression of the α-chains of FcγRI, FcγRIII, and FcγRIV (Fig. 2*B*). NOTAM macrophages ingested dual IgG- and complement C3b/iC3b-opsonized hRBCs by phagocytic cup formation or, to a lesser extent, by partial or complete sinking phagocytosis (Fig. 2*C*). Typically, phagocytic cup-like structures were generated by the extension of membrane protrusions (ruffles), which rolled over the particle (Fig. 2*C*). These protrusions were highly dynamic and susceptible to retraction. Phagocytic cup formation was seldom observed by *Fcer1g*^−/−^ macrophages, which predominantly ingested dual IgG- and complement C3b/iC3b-opsonized hRBCs *via* partial or complete sinking phagocytosis (Fig. 2*D*). This discrepancy can be gleaned from cumulative plots of phagocytic events by individual NOTAM and *Fcer1g*^−/−^ macrophages (Fig. 2*E*), which additionally indicate higher phagocytic efficiency in NOTAM macrophages than in *Fcer1g*^−/−^ macrophages (summarized in Fig. 2*F*). The lower phagocytic efficiency and lack of phagocytic cups in *Fcer1g*^−/−^ macrophages, compared with NOTAM macrophages, may be explained by relative differences in FcγRIIb activity (20). NOTAM macrophages express FcγRI, FcγRIII, and FcγRIV, each of which associates with a mutated (non-signaling ITAM) Fc receptor γ chain (21) and competes with the inhibitory Fc receptor FcγRIIb for binding to the Fc region of opsonic IgG, whereas this competitive binding is missing in *Fcer1g*^−/−^ macrophages because the other three Fcγ receptors (I, III, and IV) require the Fc receptor γ chain for surface expression (21, 22, 23). In summary, these data indicate that complement receptors mediate both sinking phagocytosis and phagocytic cups (typically emanating from extended membrane ruffles), the latter of which are susceptible to inhibition by ITIM-containing FcγRIIb, suggesting that complement receptor–mediated phagocytic cup formation may involve the activation of Syk.

### Confirmation that complement receptors ingest complement C3b/iC3b-opsonized hRBCs *via* phagocytic cup formation and sinking phagocytosis

**Figure 3:**
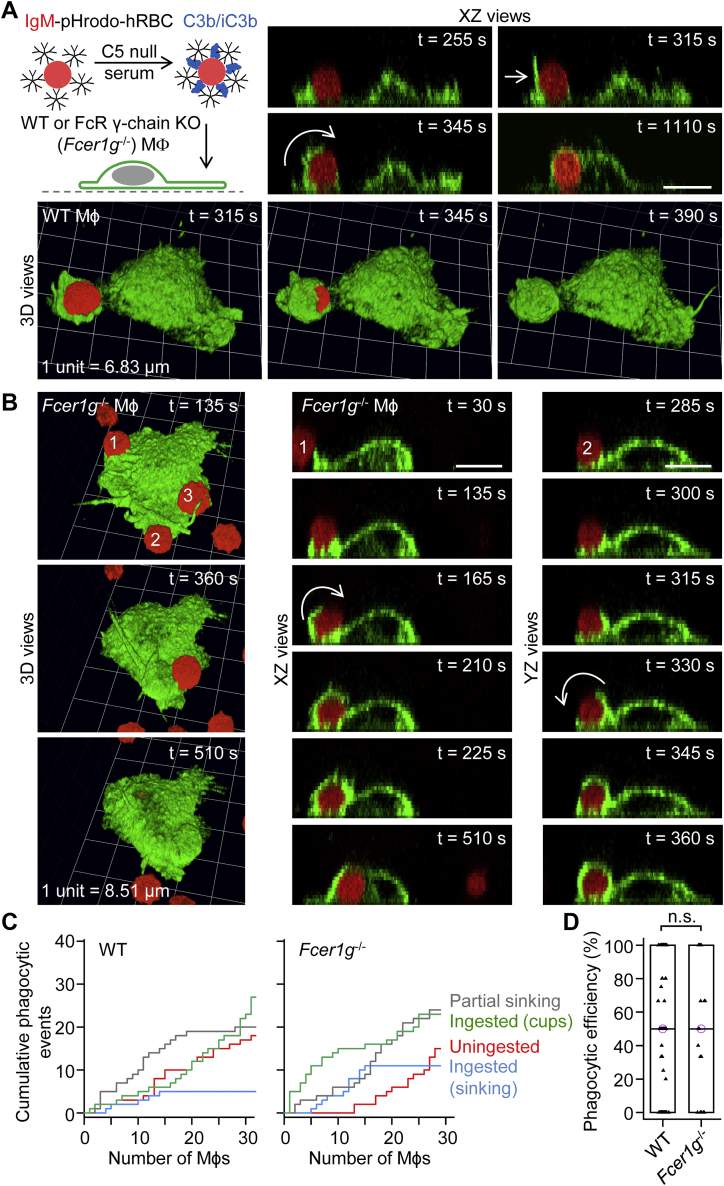
**Phagocytosis of dual IgM– and complement C3b/iC3b–opsonized human red blood cells by WT and *Fcer1g***^**−/−**^**macrophages *via* sinking or phagocytic cup formation.***A*, ingestion of a dual IgM– and complement C3b/iC3b–opsonized human red blood cell (hRBC) by a WT macrophage. The macrophage was labeled green fluorescent with Alexa Fluor 488–conjugated anti-F4/80 antibodies, and the cytosol of the hRBC was loaded with the red fluorescent probe pHrodo Red. Time-lapse 3D imaging for 30 min was performed by spinning disk confocal microscopy. Particle engulfment was achieved by a tangental membrane protrusion (*straight white arrow*), which subsequently rolled over (*curved white arrow*) the hRBC. Scale bar, 10 μm. *B*, ingestion of several dual IgM– and complement C3b/iC3b–opsonized hRBCs by a *Fcer1g*^−/−^ (Fc receptor γ-chain KO) macrophage. The *white curved arrows* indicate engulfment *via* membrane protrusions rolling over the hRBC. Scale bars, 10 μm. *C*, cumulative plots of phagocytic (ingestion) events for WT and *Fcer1g*^−/−^ macrophages. The *x*-axes show individual macrophages. *D*, summary box plots of phagocytic efficiency, defined as the percentage of dual IgM– and complement C3b/iC3b–opsonized hRBCs (in contact with a macrophage), which were fully ingested. Medians are represented by a *horizontal line* with a *lilac-colored circle*. Data were analyzed using the Mann–Whitney U test (U = 1177.5, *p* = 0.47); *n* (number of macrophages) = 92 (from 3 WT mice) and *n* = 28 (from 2 *Fcer1g*^−/−^ mice). n.s., not significant (*p* > 0.05). IgM, immunoglobulin M.

We used an alternative approach to confirm that complement receptors ingest complement C3b/iC3b-opsonized hRBCs *via* phagocytic cup formation and sinking phagocytosis. hRBCs were opsonized with IgM and incubated with complement C5 null serum to produce dual IgM– and complement C3b/iC3b–opsonized hRBCs (Fig. 3*A*). Notably, IgM, which forms pentamers in human and mouse, is not recognized by Fcγ receptors. Introduction of dual-opsonized hRBCs to WT (Fig. 3*A*; Video S3) or *Fcer1g*^−/−^ macrophages (Fig. 3*B*) sporadically induced phagocytosis *via* phagocytic cups, typically formed by the extension and rolling over of membrane protrusions (ruffles). Note that the hRBCs were loaded with the red fluorescent probe pHrodo Red, which is less susceptible to photobleaching than CMO. Ingestion of dual IgM– and complement C3b/iC3b–opsonized hRBCs by WT macrophages was considerably less efficient than the ingestion of IgG-opsonized hRBCs (compare Fig. 1*D* and Fig. 3*C*). There was no difference in the efficiency of phagocytosis (particle internalization) between WT and *Fcer1g*^−/−^ macrophages (Fig. 3*D*). In summary, these experiments, which circumvented all four Fcγ receptors, confirm that complement receptors ingest particles *via* phagocytic cup formation or sinking phagocytosis.

### Conditional deletion of Syk in macrophages abolishes FcγR-mediated phagocytosis but does not impair the complement-mediated sinking mode of phagocytosis

**Figure 4:**
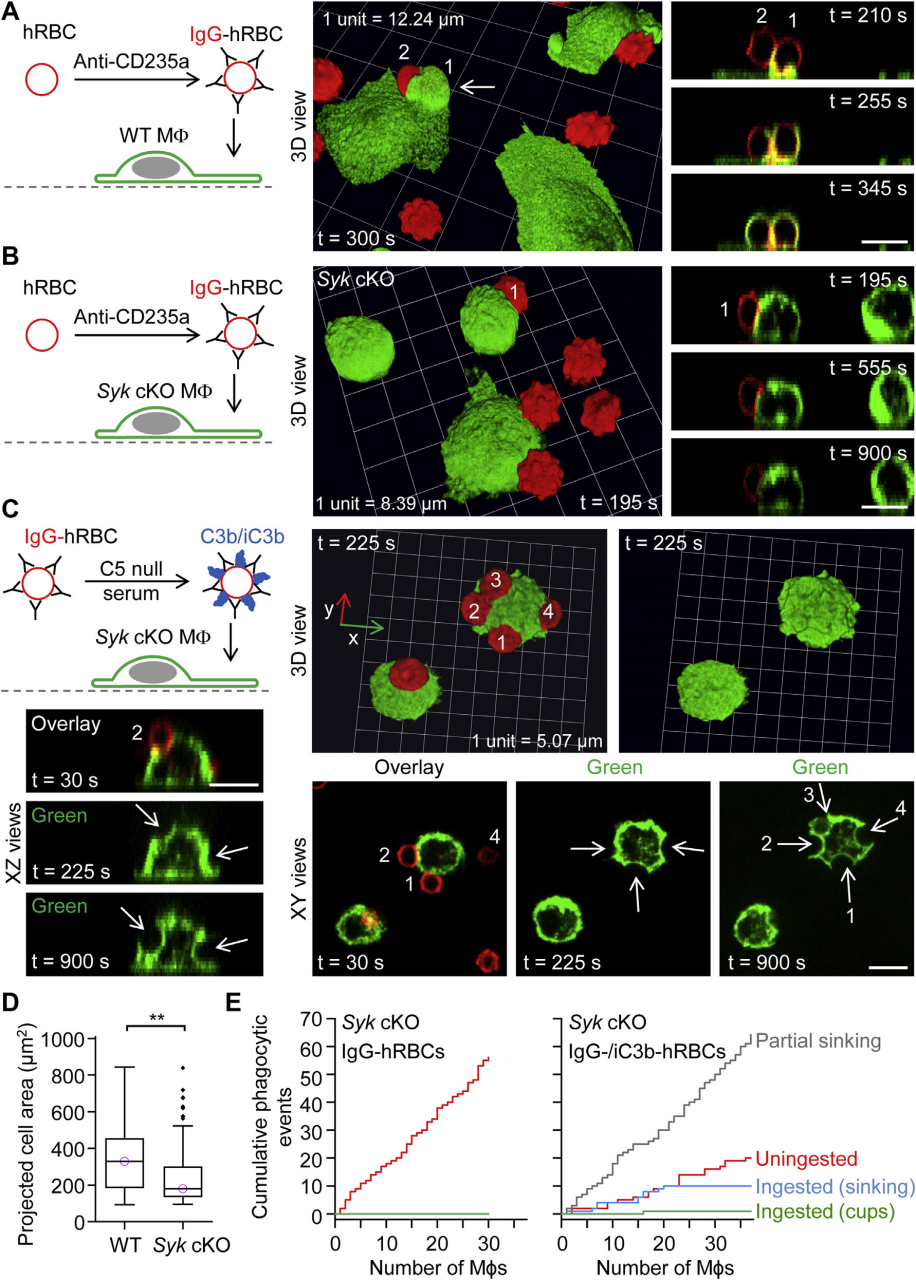
**Conditional deletion of *Syk* in macrophages inhibits Fcγ receptor–mediated phagocytosis and phagocytic cup formation by complement receptors.***A*, ingestion of a pair of IgG-opsonized human red blood cells (hRBCs) by a WT macrophage. 3D time-lapse imaging for 16 min was performed by spinning disk confocal microscopy. Macrophages were labeled green fluorescent with Alexa Fluor 488–conjugated anti-F4/80 antibodies, and the plasma membrane was labeled red fluorescent with CellMask Orange. Scale bar, 10 μm. *B*, lack of ingestion of IgG-opsonized hRBCs by macrophages isolated from myeloid-restricted *Syk* conditional KO (cKO) mice. Scale bar, 10 μm. *C*, partial sinking of dual IgM– and complement C3b/iC3b–opsonized hRBCs into *Syk* cKO macrophages. The *white arrows* indicate sinking phagocytic events. Scale bars, 10 μm. *D*, summary box plots of projected cell area in WT and *Syk* cKO macrophages. The medians are represented by a *horizontal line* with a *lilac-colored circle*. Data were analyzed using the Mann–Whitney U test (U = 6896, *p* =0.0004); *n* (number of macrophages) = 122 (from 4 WT mice) and *n* = 88 (from 3 *Syk* cKO mice). ∗∗*p* < 0.001. *E*, cumulative plots of phagocytic (ingestion) events for *Syk* cKO macrophages presented with either IgG-opsonized or dual IgG– and complement C3b/iC3b–opsonized hRBCs. The *x*-axes show individual macrophages. IgG, immunoglobulin G.

In contrast to WT macrophages (Fig. 4*A*; Videos S4–S6), macrophages isolated from myeloid-restricted *Syk* conditional KO (cKO) mice did not ingest IgG-opsonized hRBCs (Fig. 4*B*; Video S7). Notably, Video S4 and the 3D reconstruction in Video S5 show a nice example of the tip of an hRBC being pinched off by cup closure. Neither phagocytic cup formation nor partial sinking phagocytosis was observed in *Syk* cKO macrophages (Fig. 4*B*). This suggests that Zap70 (encoded by *Zap70*), which was not expressed in WT peritoneal F4/80^+^ cells, does not compensate for loss of Syk. Consistent with this interpretation, Mócsai *et al*. (24) did not detect Zap70 protein in either WT or Syk KO neutrophils. In contrast to singly IgG-opsonized hRBCs, dual IgG- and complement C3b/iC3b-opsonized hRBCs, stained with CMO, appeared to slowly sink into *Syk* cKO macrophages (Fig. 4*C*). Moreover, *Syk* cKO macrophages exhibited less cell spreading, indexed as projected 2D area, than WT macrophages (Fig. 4*D*). Cell spreading was assessed after overnight incubation on fibronectin-coated surfaces. Cumulative plots of phagocytic events by individual *Syk* cKO macrophages (Fig. 4*E*) highlight, on the one hand, the complete loss of the ability to ingest IgG-opsonized hRBCs, and, on the other, persistent partial or complete ingestion of complement-opsonized hRBCs *via* sinking phagocytosis in the absence of the tyrosine kinase Syk.

**Figure 5:**
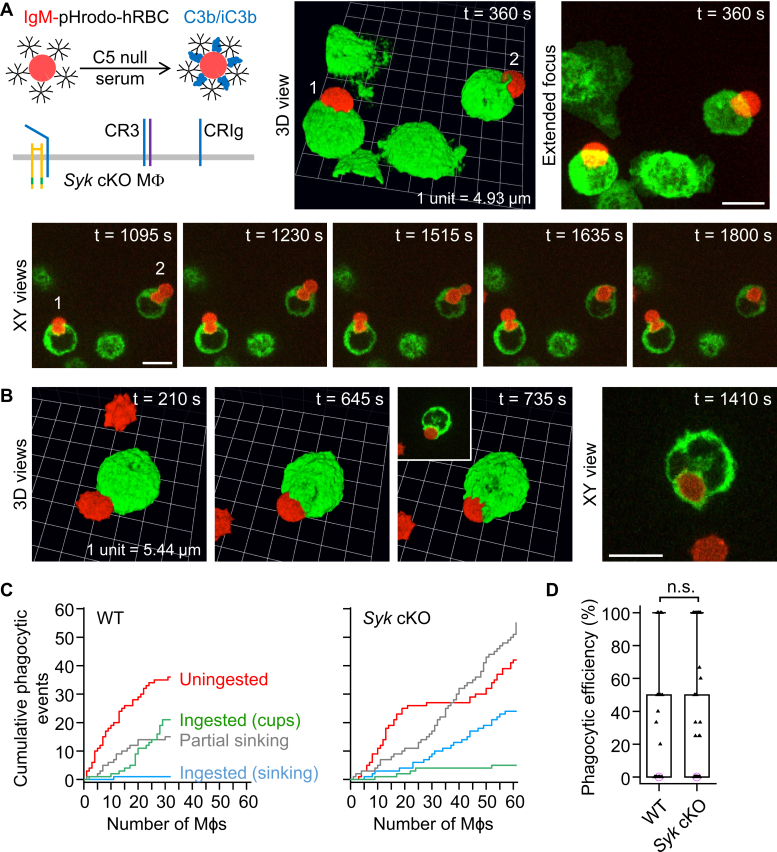
**Slow sinking phagocytosis mediated by complement receptors in *Syk* conditional KO (cKO) macrophages presented with dual IgM– and complement C3b/iC3b–opsonized targets.***A* and *B*, partial or complete ingestion of dual IgM– and complement C3b/iC3b–opsonized human red blood cells (hRBCs) by myeloid-restricted *Syk* conditional KO (cKO) macrophages. The *inset* in the lower panel (*B*) shows an XY optical section. 3D time-lapse imaging was performed by spinning disk confocal microscopy. Macrophages were labeled green fluorescent with Alexa Fluor 488–conjugated anti-F4/80 antibodies, and the cytosol of hRBCs was loaded with the red fluorescent probe pHrodo Red. Scale bars, 10 μm. *C*, cumulative plots of phagocytic (ingestion) events for WT and *Syk* cKO macrophages presented with dual IgM– and complement C3b/iC3b–opsonized hRBCs. The *x*-axes show individual macrophages. *D*, summary box plots of phagocytic efficiency, defined as the percentage of dual IgM– and complement C3b/iC3b–opsonized hRBCs (in contact with a macrophage), which were fully ingested. Medians are represented by a *horizontal line* with a *lilac-colored circle*. Data were analyzed using the Mann–Whitney U test (U = 1002, *p* =0.6); *n* (number of macrophages) = 31 (from 3 WT mice) and *n* = 61 (from 3 *Syk* cKO mice). n.s. = not significant (*p* > 0.05). IgM, immunoglobulin M.

Consistent with the above observations, we found that dual IgM– and complement C3b/iC3b–opsonized hRBCs, loaded with pHrodo Red, were partially or fully ingested by *Syk* cKO macrophages *via* sinking phagocytosis (Fig. 5, *A* and *B*; Videos S8 and S9). During sinking phagocytosis, the hRBCs were typically squeezed through a narrow orifice, giving rise to intermediary hourglass-shaped hRBCs (lower panel in Fig. 5*A*; Video S8). After passage through the restricted point of entry, the phagosome adopted a rounded morphology. Cumulative plots indicate that dual IgM–opsonized and C3b/iC3b-opsonized hRBCs were often uningested in both WT and *Syk* cKO macrophages (Fig. 5*C*). In the case of ingested particles, individual WT macrophages frequently internalized complement-opsonized hRBCs *via* phagocytic cup formation (Fig. 5*C*, left plot), whereas complement-opsonized particles were predominantly internalized by sinking phagocytosis in *Syk* cKO macrophages (Fig. 5*C*, right plot). Thus, loss of phagocytic cup formation by *Syk* cKO macrophages was compensated by increased sinking phagocytosis, and there was no significant difference in the median phagocytic efficiency (Fig. 5*D*). In summary, the data shown in Figures 4 and 5 indicate that Syk is required for both FcγR-mediated and complement receptor–mediated phagocytic cup formation, and, in the absence of Syk, the distinctive complement receptor–mediated sinking mode of phagocytosis prevails.

### ITAM adaptors are required for complement receptor–mediated phagocytic cup formation

**Figure 6:**
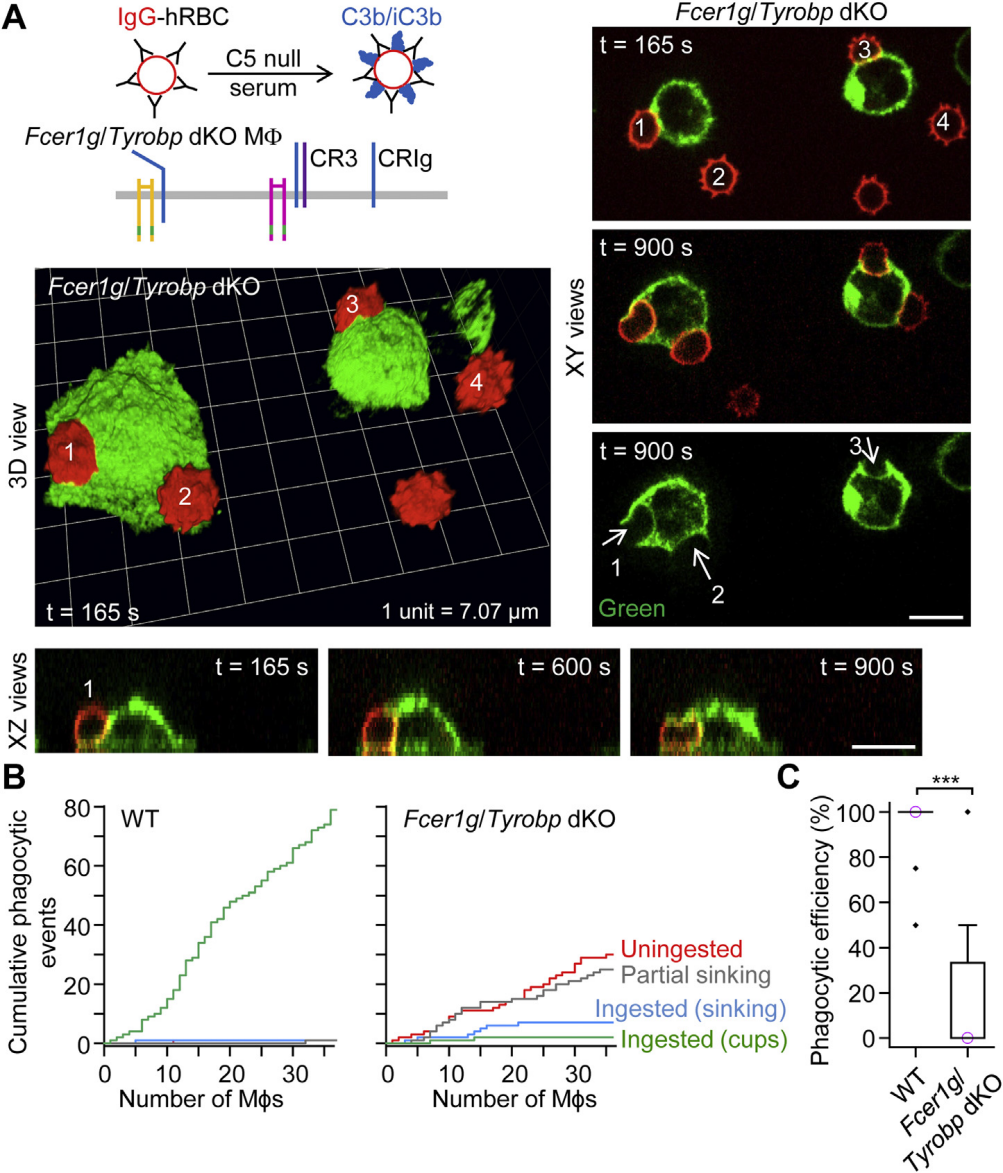
**Slow sinking phagocytosis in macrophages lacking the ITAM adaptor Fc receptor γ chain and DAP12.***A*, slow partial sinking of dual IgG– and complement C3b/iC3b–opsonized human red blood cells (hRBCs) into *Fcer1g*/*Tyrobp* double KO (dKO) macrophages (Mϕs), which lack the ITAM adaptor proteins Fc receptor γ chain and DAP12. Time-lapse 3D imaging for 16 min was performed by spinning disk confocal microscopy. Macrophages were labeled green fluorescent with Alexa Fluor 488–conjugated anti-F4/80 antibodies, and the plasma membrane was labeled red fluorescent with CellMask Orange. Scale bars, 10 μm. *B*, cumulative plots of phagocytic (ingestion) events for WT and *Fcer1g*/*Tyrobp* dKO macrophages presented with dual IgG– and complement C3b/iC3b–opsonized hRBCs. The *x*-axes show individual macrophages. *C*, summary box plots of phagocytic efficiency, defined as the percentage of dual IgG– and complement C3b/iC3b–opsonized hRBCs (in contact with a macrophage) which were fully ingested. Medians are represented by a *horizontal line* with a *lilac-colored circle*. Data were analyzed using the Mann–Whitney U test (U = 1465.5, *p* < 0.0001); *n* (number of macrophages) = 46 (from 2 WT mice) and *n* = 35 (from 2 *Fcer1g*/*Tyrobp* dKO mice). ∗∗∗*p* < 0.0001. IgG, immunoglobulin G; ITAM, immunoreceptor tyrosine-based activation motif.

We next asked how complement receptors induce Syk-dependent phagocytic cup formation. We hypothesized that CR3, a β_2_ integrin, associates with the Fc receptor γ chain and/or DAP12 to induce Syk-dependent phagocytic cup formation because integrin signaling was previously reported by Mocsai *et al*. (25) to involve these ITAM-containing adaptor proteins. Consistent with this hypothesis, macrophages lacking both *Fcer1g* (Fc receptor γ chain) and *Tyrobp* (DAP12) slowly ingested dual IgG– and complement C3b/iC3b–opsonized hRBCs by sinking phagocytosis, with a paucity of membrane protrusive activity (Fig. 6*A*). Furthermore, similar to *Syk* cKO macrophages, *Fcer1g*/*Tyrobp* double KO (dKO) macrophages exhibited cell spreading defects. Cumulative plots of the response of individual macrophages to dual IgG- and C3b/iC3b-opsonized hRBCs show that WT macrophages ingested particles exclusively by phagocytic cup formation, whereas phagocytic cup formation was negligible in *Fcer1g*/*Tyrobp* dKO macrophages. Instead, *Fcer1g*/*Tyrobp* dKO macrophages partially or fully ingested dual IgG- and complement-opsonized hRBCs *via* sinking phagocytosis.

**Figure 7:**
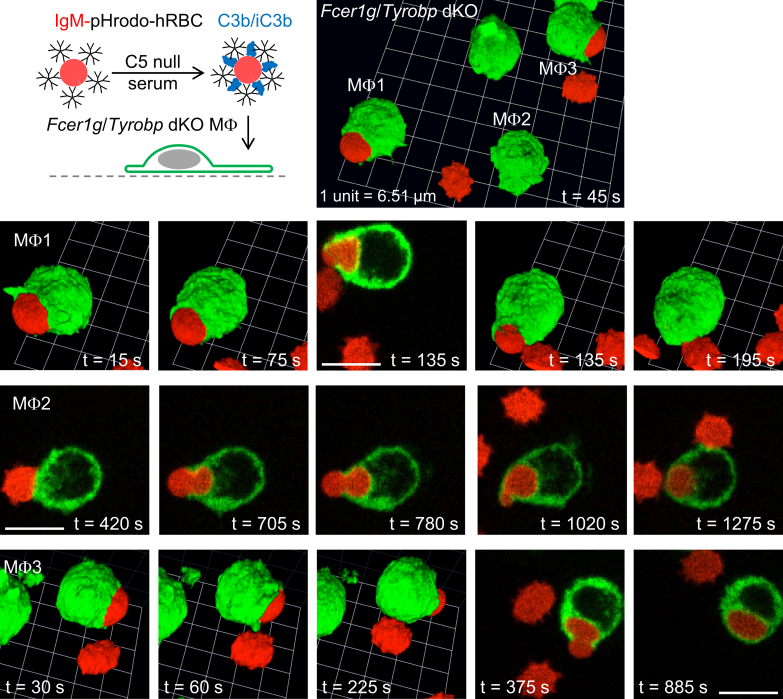
**Ingestion of dual IgM– and C3b/iC3b–opsonized human red blood cells by *Fcer1g*/*Tyrobp* double KO (dKO) macrophages.** Ingestion of dual IgM– and complement C3b/iC3b–opsonized human red blood cells (hRBCs) by *Fcer1g*/*Tyrobp* dKO macrophages (Mϕs), which lack the ITAM-containing Fc receptor γ chain and the ITAM adaptor DAP12. Time-lapse 3D imaging for 30 min was performed by spinning disk confocal microscopy. Macrophages were labeled green fluorescent with Alexa Fluor 488–conjugated anti-F4/80 antibodies, and the cytosol of hRBCs was loaded with the red fluorescent probe pHrodo Red. Scale bars, 10 μm. IgM, immunoglobulin M; ITAM, immunoreceptor tyrosine-based activation motif.

Using hRBCs dual opsonized with IgM and complement C3b/iC3b, we confirmed that *Fcer1g*/*Tyrobp* dKO macrophages have impaired lamellipodial cell spreading and phagocytic cup formation, but slowly ingest complement-opsonized particles by sinking phagocytosis (Fig. 7; Videos S10–S12). Similar to the case with *Syk* cKO macrophages, dual-opsonized hRBCs were internalized into *Fcer1g*/*Tyrobp* dKO macrophages by squeezing through a narrow orifice, producing hourglass-like deformities (Fig. 7; Videos S10–S12). In summary, the data shown in Figures 6 and 7 indicate that phagocytic complement receptors use ITAM adaptors to generate phagocytic cups, and in the absence of these adaptors, slow sinking phagocytosis persists.

### Deletion of Tyrobp (DAP12) accentuates sinking phagocytosis *via* complement receptors

**Figure 8:**
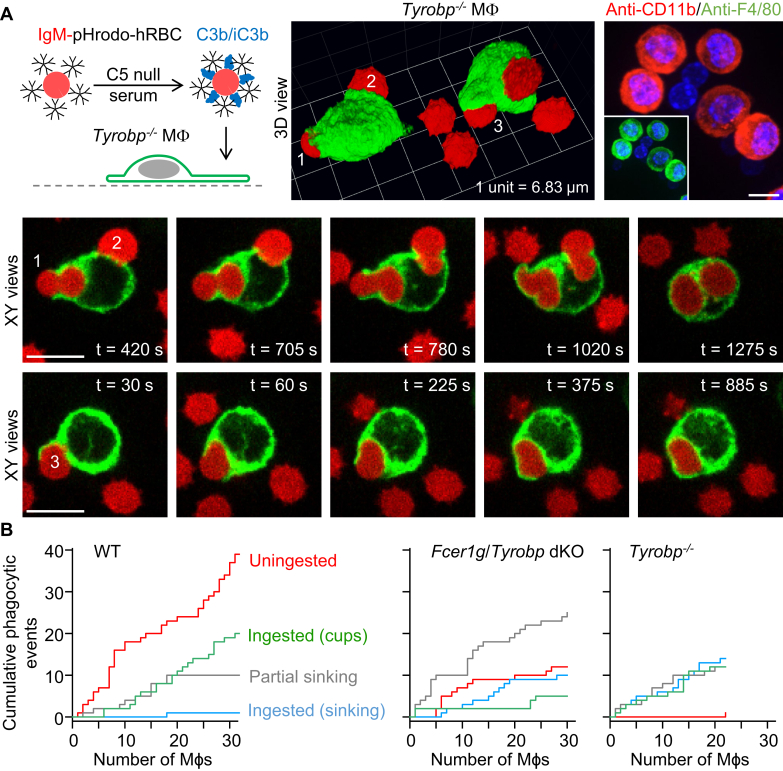
**Phagocytic cup formation is less impaired in macrophages lacking *Tyrobp* (DAP12) than in those lacking both *Tyrobp* and *Fcer1g*.***A*, time-lapse 3D images obtained by spinning disk confocal microscopy showing slow sinking phagocytosis events associated with various degrees of membrane protrusive activity. *Tyrobp* (DAP12) KO (*Tyrobp*^−/−^) macrophages (Mϕs) were presented with dual IgM– and C3b/iC3b–opsonized human red blood cells (hRBCs). Macrophages were labeled green fluorescent with Alexa Fluor 488–conjugated anti-F4/80 antibodies, and the cytosol of hRBCs was loaded with the red fluorescent probe pHrodo *Red*. The extended focus image at the top right shows resident peritoneal *Tyrobp*^−/−^ cells labeled with Alexa Fluor 594–conjugated anti-CD11b (CD11b is a component [α-subunit] of complement receptor 3) and Alexa Fluor 488–conjugated anti-F4/80 antibodies (*inset*). Nuclei (*blue channel*) were stained with Hoechst 33342, a fluorescent nucleic acid stain. Scale bars, 10 μm. *B*, cumulative plots of phagocytic (ingestion) events for WT, *Fcer1g*/*Tyrobp* double KO (dKO), and *Tyrobp*^−/−^ macrophages presented with dual IgM– and complement C3b/iC3b–opsonized hRBCs. The *x*-axes show individual macrophages. IgM, immunoglobulin M.

We speculated that CR3 may preferentially recruit DAP12 rather than the Fc receptor γ chain. Immunofluorescence staining confirmed that both components of CR3, the α-chain (CD11b) and the β-chain (CD18; not shown), are expressed in *Tyrobp*^−/−^ (DAP12 KO) macrophages, counterstained with anti-F4/80 antibodies (Fig. 8*A*). Similar to *Syk* cKO and *Fcer1g*/*Tyropb* dKO macrophages, *Tyrobp*^−/−^ macrophages presented with dual IgM– and complement C3b/iC3b–opsonized hRBCs exhibited impaired lamellipodial cell spreading, whereas sinking phagocytosis prevailed (Fig. 8*A*; Videos S13 and S14). However, phagocytic cup formation by *Tyrobp*^−/−^ macrophages was less impaired than dKO mutants (Fig. 8*B*). As observed in macrophages isolated from *Syk* cKO and *Fcer1g*/*Tyrobp* dKO mice, *Tyrobp*^−/−^ macrophages exhibited slow sinking phagocytosis with varying degrees of membrane protrusive activity. The bottom panel in Figure 8*A* (Videos S14) shows an example in which particle internalization is achieved by a combination of sinking phagocytosis and an enfolding membrane extension. In summary, these data indicate that complement receptor–mediated phagocytic cup formation requires recruitment of Syk by either of the two ITAM adaptors, DAP12 and Fc receptor γ chain.

### Complement-induced phagocytic cup formation and sinking phagocytosis are clearly mediated by CR3

**Figure 9:**
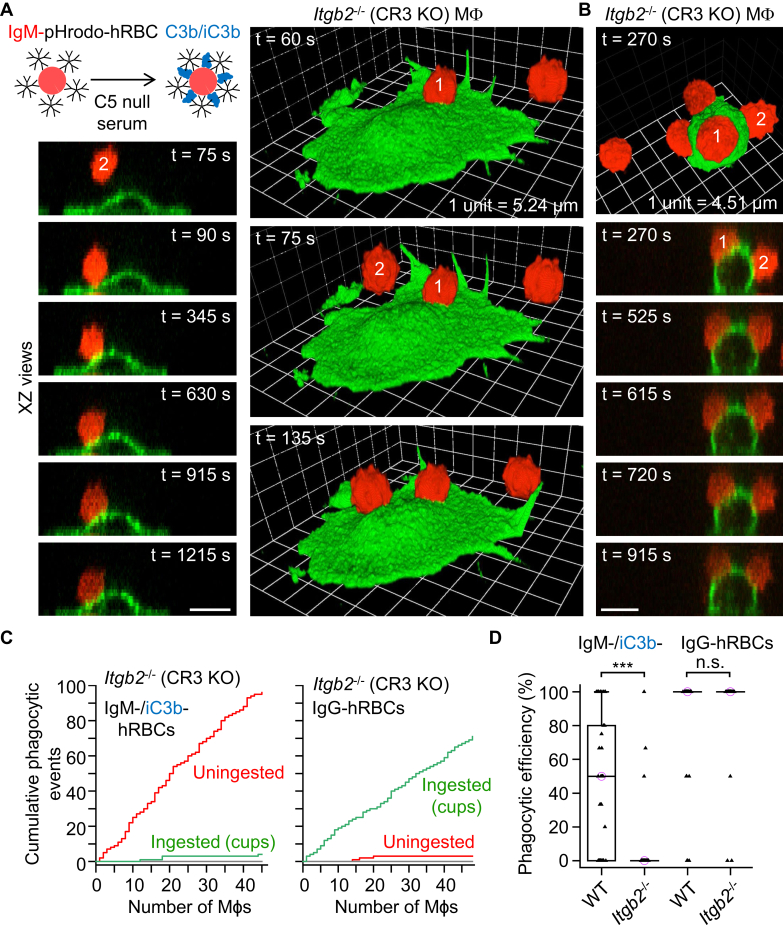
**Phagocytosis of dual IgM– and C3b/iC3b–opsonized human red blood cells is abrogated in *Itgb2***^**−/−**^**(complement receptor 3 [CR3] KO) macrophages.***A* and *B*, 3D time-lapse images obtained by spinning disk confocal microscopy showing lack of phagocytic activity in *Itgb2*^−/−^ (CR3 KO) macrophages, irrespective of whether cells were spread out (*A*) or rounded up (*B*). *Itgb2*^−/−^ (CR3 KO) macrophages (Mϕs) were presented with dual IgM– and complement C3b/iC3b–opsonized human red blood cells (hRBCs). Macrophages were labeled green fluorescent with Alexa Fluor 488–conjugated anti-F4/80 antibodies, and the cytosol of hRBCs was loaded with the red fluorescent probe pHrodo Red. Scale bars (*A* and *B*), 10 μm. *C*, cumulative plots of phagocytic (ingestion) events for *Itgb2*^−/−^ macrophages presented with either dual IgM– and complement C3b/iC3b–opsonized hRBCs or IgG-opsonized hRBCs. *D*, summary box plots of phagocytic efficiency, defined as the percentage of dual IgM– and complement C3b/iC3b–opsonized hRBCs or IgG-opsonized hRBCs (in contact with a macrophage), which were fully ingested. Medians are represented by a *horizontal line* with a *lilac-colored circle*. Data were analyzed using the Kruskal–Wallis one-way ANOVA (H = 121.8, degrees of freedom = 3, and *p* < 0.0001). Post hoc comparisons were performed using the Dunn test; *n* (number of macrophages) = 46 (from 3 WT mice), *n* = 44 (from 3 CR3 KO [*Itgb2*^−/−^] mice), *n* = 52 (from 3 WT mice), and *n* = 48 (from 3 CR3 KO mice), respectively, from left to right. ∗∗∗*p* < 0.0001. IgM, immunoglobulin M; IgG, immunoglobulin G.

Up to this point, we have not elucidated whether one or both of the complement receptors expressed in peritoneal F4/80^+^ cells (macrophages), CR3 and CRIg, mediate complement C3b/iC3b-induced phagocytic cup formation and sinking phagocytosis. We challenged *Itgb2*^−/−^ macrophages, which lack the β_2_-chain (also known as CD18) and surface expression of CR3, with dual IgM– and complement C3b/iC3b–opsonized hRBCs (Fig. 9, *A* and *B*). Neither phagocytic cup formation nor sinking phagocytosis was observed in either spread out (Fig. 9*A*) or unspread (Fig. 9*B*) *Itgb2*^−/−^ (CR3 KO) macrophages. Cumulative plots show that *Itgb2*^−/−^ macrophages largely fail to ingest dual IgM– and complement C3b/iC3b–opsonized hRBCs, and, importantly, sinking phagocytosis is not observed, whereas IgG-opsonized hRBCs were ingested by phagocytic cup formation (Fig. 9*C*). The phagocytic (complete internalization) efficiencies of WT and *Itgb2*^−/−^ macrophages are compared in Figure 9*D*. In summary, these data indicate that CR3 is essential for complement-induced phagocytic cup formation and sinking phagocytosis. CRIg may be involved in particle tethering (26), but it cannot compensate for loss of CR3-mediated phagocytic activity.

### FcγR-mediated phagocytic cups become highly elongated and cylindrical in the absence of CR3

**Figure 10:**
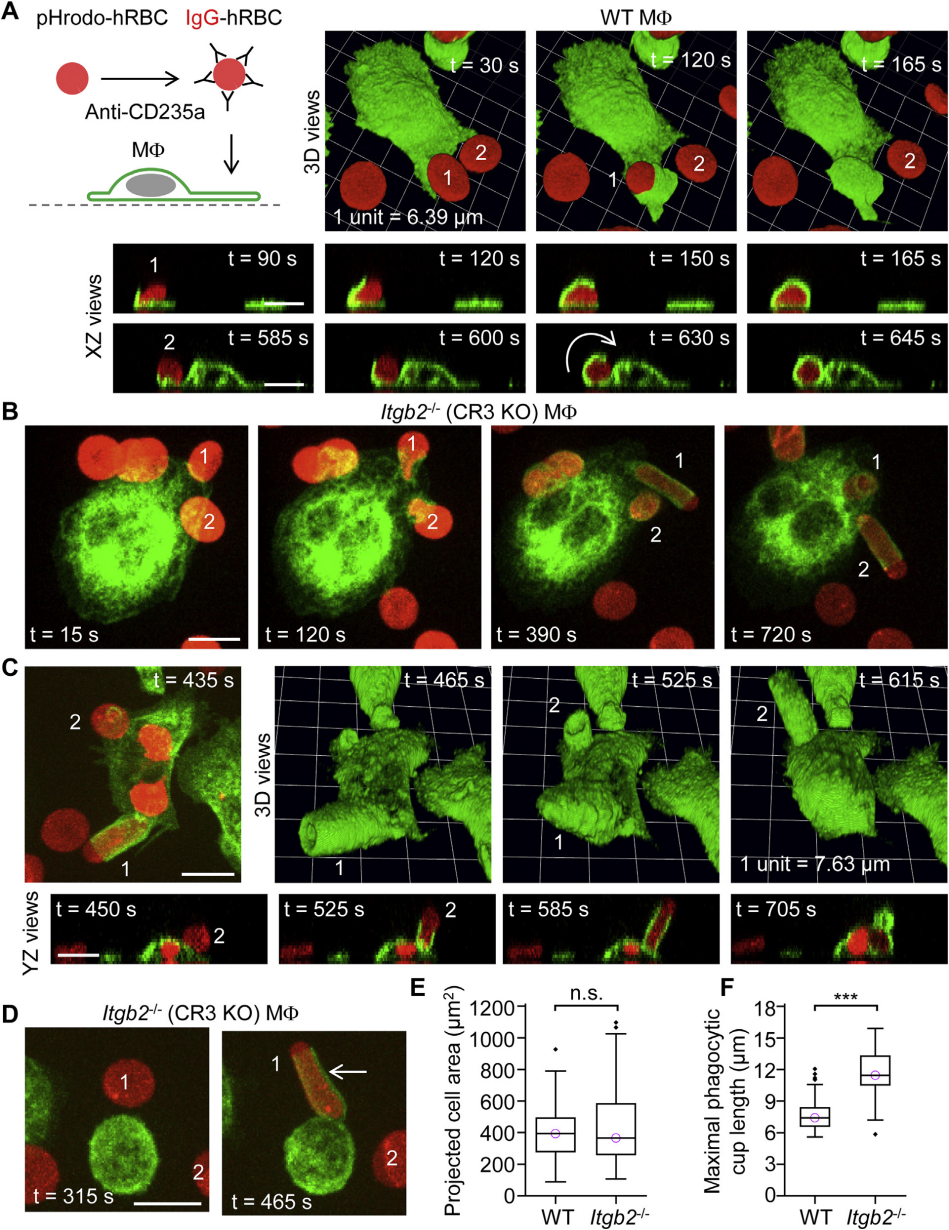
**Aberrant elongated and cylindrical Fcγ receptor–mediated phagocytic cups in *Itgb2***^**−/−**^**(complement receptor 3 [CR3] KO) macrophages.***A*, ingestion of an IgG-opsonized human red blood cell (hRBC) by a WT macrophage. 3D time-lapse imaging was performed by spinning disk confocal microscopy. Macrophages were labeled green fluorescent with Alexa Fluor 488–conjugated anti-F4/80 antibodies and the cytosol of hRBCs was loaded with the red fluorescent probe pHrodo Red. Scale bars, 10 μm. *B* and *C*, *Itgb2*^−/−^ (CR3 KO) macrophages ingest IgG-opsonized hRBCs *via* markedly elongated, cylindrical phagocytic cups. Scale bars, 10 μm. *D*, elongated, cylindrical phagocytic cup produced by a morphologically spherical *Itgb2*^−/−^ macrophage. Scale bar, 10 μm. *E*, summary box plots of projected cell area in WT and *Itgb2*^−/−^ macrophages. Medians are represented by a *horizontal line* with a *lilac-colored circle*. Data were analyzed using the Mann–Whitney U test (U = 3015, *p* =0.69); *n* (number of macrophages) = 55 (from 3 WT mice) and *n* = 114 (from 3 *Itgb2*^−/−^ mice). n.s., not significant (*p* > 0.05). *F*, summary box plots of the maximal phagocytic cup length in WT and *Itgb2*^−/−^ macrophages. Data were analyzed using the Mann–Whitney U test (U = 290, *p* < 0.0001); *n* (number of macrophages) = 62 (from 3 WT mice) and *n* = 56 (from 3 *Itgb2*^−/−^ mice). ∗∗∗*p* < 0.0001. IgG, immunoglobulin G.

Although the β_2_-integrin CR3 has been variously implicated in FcγR-mediated functions (27, 28, 29), we did not expect deletion of CR3 to dramatically affect FcγR-mediated phagocytic cup formation. In contrast to WT macrophages, which modestly deformed IgG-opsonized hRBCs during engulfment (Fig. 10*A*), *Itgb2*^−/−^ (CR3 KO) macrophages ingested IgG-opsonized hRBCs with exceedingly long and cylindrical phagocytic cups (Fig. 10*B*; Video S15). Aberrant, elongated phagocytic cups were most obvious when they were formed parallel to the XY plane, but 3D time-lapse imaging revealed that these structures commonly pivot at the base to assume more upright positions before becoming slowly internalized (Fig. 10, *B* and *C*). Elongated, cylindrical cups could also be observed extending from otherwise morphologically spherical macrophages (Fig. 10*D*). Cell spreading, assessed after overnight incubation on fibronectin-coated channel slides, was not impaired in *Itgb2*^−/−^ macrophages, which lack the fibronectin-binding β_2_-integrin CR3 (Fig. 10*E*), but express other fibronectin-binding integrins, such as β_1_α_4_. The maximal phagocytic cup lengths, measured using XY, XZ, and YZ views, for WT and *Itgb2*^−/−^ macrophages are shown in Figure 10*F*. In summary, these data underscore that CR3 is intimately involved in FcγR-mediated phagocytic cup dynamics and closure, and loss of CR3 leads to, at least in the case of highly deformable particles, strikingly elongated phagocytic cups.

**Figure 11:**
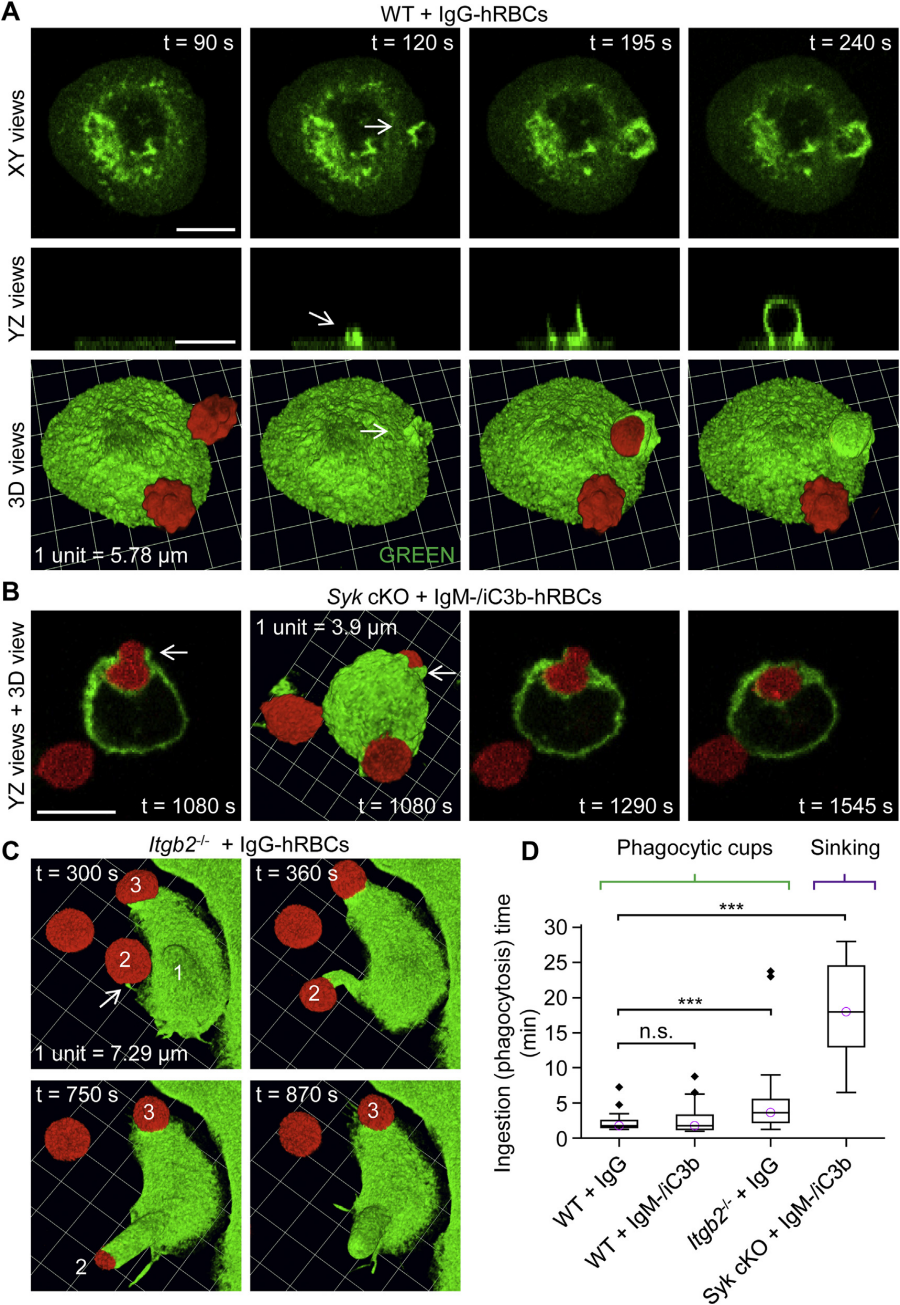
**Kinetics of phagocytic cup formation and sinking phagocytosis.***A*, ingestion of an IgG-opsonized human red blood cell (hRBC) by a WT macrophage. 3D time-lapse imaging was performed by spinning disk confocal microscopy. Macrophages were labeled green fluorescent with Alexa Fluor 488–conjugated anti-F4/80 antibodies, and the plasma membrane was labeled *red* fluorescent with CellMask Orange. Note that the contact point between the IgG-opsonized hRBC and the macrophage elicits local membrane protrusions, which mark the initiation of phagocytic cup formation. Scale bars, 10 μm. *B*, ingestion of dual IgM– and complement C3b/iC3b–opsonized hRBCs by a *Syk* cKO macrophage *via* slow sinking phagocytosis. Local membrane protrusions (indicated by *white arrows*) accompany sinking phagocytosis to varying extents. Scale bar, 10 μm. *C*, formation of a phagocytic cup upon a filopodium, extending from an *Itgb2*^−/−^ (complement receptor 3 deficient) macrophage. The *white arrow* shows thickening of the membrane at the tip of a filopodium, where it makes contact with an IgG-opsonized hRBC. The bulge of the macrophage cell body, marked by the *white numerical digit 1*, indicates where an IgG-opsonized hRBC has been fully internalized. *D*, summary box plots of ingestion times for phagocytic cup formation and sinking phagocytosis. Medians are represented by a *horizontal line* with a *lilac-colored circle*. hRBCs were singly opsonized with IgG or dual-opsonized with IgM and complement C3b/iC3b, as indicated. Data were analyzed using the Kruskal–Wallis one-way ANOVA (H = 81.3, degrees of freedom = 3, and *p* < 0.0001). Post hoc comparisons were performed using the Dunn test; *n* (number of macrophages) = 59 (from 6 WT mice), *n* = 37 (from 3 WT mice), *n* = 52 (from 3 *Itgb2*^−/−^ mice), and *n* = 23 (from 3 *Syk* cKO mice), respectively, from left to right. ∗∗∗*p* < 0.0001. IgM, immunoglobulin M; IgG, immunoglobulin G.

As in the case of phagocytic cup length, the duration of phagocytic cup formation or sinking phagocytosis was determined using XY, XZ, and XY views of 3D time-lapse recordings (Fig. 11). The initiation of phagocytic cup formation could be readily identified as dorsal membrane thickenings on lamellipodial membrane extensions, as shown in XY, YZ, and 3D reconstruction views (Fig. 11*A*). Phagocytic cups commonly extended on one side of a particle and thereby developed a hood-like, rather than cup-like, shape, as in the examples in Figures 2*C*, 4*A*, and 11*A*. The initiation of sinking phagocytosis was less obvious, although complete internalization was much slower, or became stalled, and sinking was accompanied by varying degrees of membrane-protrusive activity around the portal of entry, as shown in Figure 11*B*, possibly driven by the activity of Src-family kinases (30). FcγR-mediated phagocytic cups could emerge from membrane protrusions, including filopodia, as shown in the example for an *Itgb2*^−/−^ macrophage in Figure 11*C*. In this example, the initiation of phagocytosis is marked by thickening of the tip of a filopodium. Filopodia were similarly observed to initiate phagocytosis *via* FcγRs in WT macrophages, reminiscent of our previous observations with zymosan particles (31). A summary plot of phagocytosis durations is shown in Figure 11*D*. Notably, the total time for the formation and closure of elongated phagocytic cups by *Itgb2*^−/−^ macrophages was modestly longer than for more conventional phagocytic cups, and sinking phagocytosis was considerably slower than the phagocytic cup formation.

## Discussion

**Figure 12:**
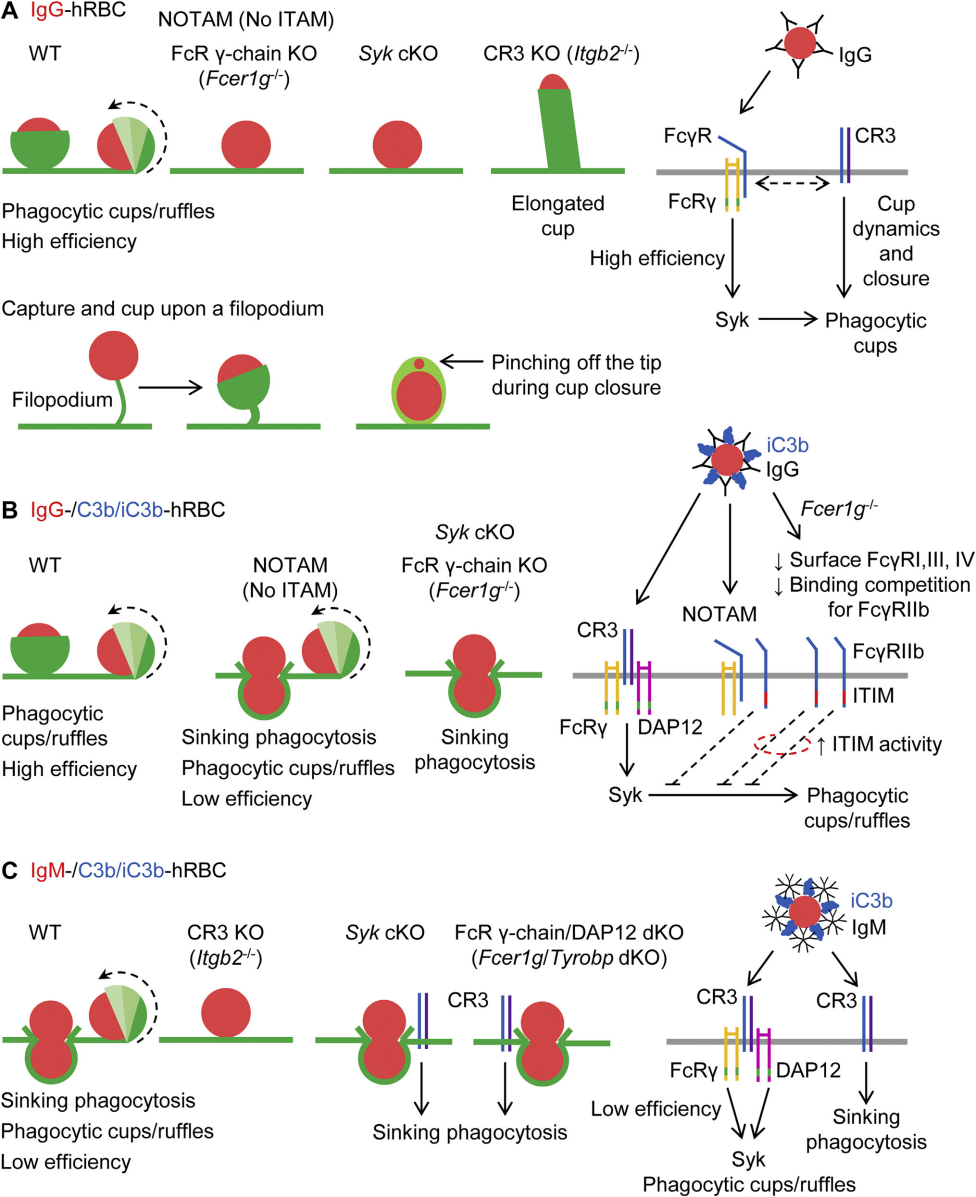
**Schematic summary.***A*, WT macrophages ingest IgG-opsonized human red blood cells (IgG-hRBCs) with high efficiency *via* rapid (2–3 min) phagocytic cup formation, which includes extension of ruffles, especially peripheral ruffles. Phagocytic cup formation after application of IgG–RBCs is abrogated in both *Fcer1g*^−/−^ macrophages, which lack the Fc receptor γ chain, and NOTAM macrophages, which express a mutant Fc receptor γ chain with a nonsignaling ITAM. Phagocytic cups are also lacking in *Syk* conditional KO (cKO) macrophages. In contrast, phagocytic cups become elongated and cylindrical in *Itgb2*^−/−^ (complement receptor 3 [CR3] KO) macrophages presented with IgG–hRBCs. A model of the signal transduction underlying Fcγ receptor (FcγR)–mediated phagocytosis is shown on the *right*. Note that phagocytic cups can form upon a filopodium and cup closure can pinch off the tips of cells, as shown in the lower panel. *B*, whereas FcγRI, FcγRIII, and FcγRIV associate with the FcR γ chain, which contains an ITAM, the inhibitory FcγR (FcγRIIb) contains an immunoreceptor tyrosine-based inhibition motif (ITIM) in its cytosolic tail. Phagocytic cup formation was less efficient in NOTAM macrophages (which have surface expression of all four FcγRs, but lack a functional ITAM) than WT macrophages, but largely blocked in *Fcer1g*^−/−^ (FcR γ chain KO) macrophages, which do not express surface FcγRI, FcγRIII, and FcγRIV. Thus, in *Fcer1g*^−/−^ macrophages, FcγRIIb has less competition for binding to opsonic IgG, which would promote ITIM signaling, as shown in the model on the *right*. *C*, WT macrophages ingest dual IgM– and complement C3b/iC3b–opsonized hRBCs *via* phagocytic cup formation, extended ruffles, and varying degrees of sinking. Dual IgM– and C3b/iC3b–opsonized hRBCs are not ingested by *Itgb2*^−/−^ (CR3 KO) macrophages, implying that CR3, rather than CRIg, induces phagocytic cup formation and sinking phagocytosis. Notably, IgM, in contrast to IgG, is not recognized by Fcγ receptors. CR3-mediated phagocytic cup formation, but not sinking phagocytosis, is abrogated in macrophages lacking Syk (*Syk* conditional KO [cKO] macrophages) or the immunoreceptor tyrosine-based activation motif (ITAM) adaptors FcR γ chain and DAP12 (*Fcer1g*/*Tyrobp* double KO [dKO] macrophages). The endocytic pathway during sinking phagocytosis is restricted in diameter and hRBCs, which are highly deformable, are squeezed into the cell interior, producing a characteristic intermediary hourglass morphology. A model of the signal transduction underlying CR3-mediated phagocytosis is shown on the *right*. IgG, immunoglobulin G; IgM, immunoglobulin M; ITAM, immunoreceptor tyrosine-based activation motif; NOTAM, No ITAM.

On close inspection of the literature since the milestone study of Kaplan (5), the mechanism of CR3-mediated phagocytosis remains very unclear. There is little evidence for CR3-mediated sinking phagocytosis, and most authors tend to cite the original Kaplan study (5) or a review article (7, 8, 9, 10, 11, 32, 33) when referring to this putative mode of phagocytosis. To clarify whether there is a sinking mode, we performed high-resolution, 3D time-lapse imaging under conditions in which the phagocytosis is unambiguously mediated by CR3. We found that there is indeed a distinct CR3-mediated sinking mode of phagocytosis, schematically illustrated in Figure 12. This sinking mode was not observed in the recent time-lapse imaging study by Jaumouillé *et al*. (15), who proposed a model in which complement receptors link actin filaments to particles to drive phagocytic membrane protrusions (34), analogous to integrin-mediated force coupling during leukocyte migration (35) and consistent with earlier work showing that integrins can act as clutches to decrease the (retrograde) flow of actin filaments toward the nucleus (36, 37). We found that sinking phagocytosis is slow and inefficient. Particle sinking was accentuated by deletion of Syk or the ITAM adaptors FcR γ chain and DAP12, whereas it was completely inhibited by deletion of the β_2_-chain of CR3 (Fig. 12). We did not explore the mechanism of sinking, but we assume that it involves the sequence of events delineated by Allen and Aderem (38), in which the β_2_-integrin CR3 links complement-opsonized particles to filamentous actin *via* adaptor proteins such as talin, vinculin, paxillin, and α-actinin.

In addition to sinking phagocytosis, CR3 also promoted phagocytic cups, albeit inefficiently, *via* the extension of membrane protrusions (ruffles), which was largely blocked by deletion of Syk or the ITAM adaptors FcR γ chain and DAP12. Deletion of Syk or ITAM adaptors also impaired lamellipodial cell spreading. We presume that Syk signaling induces both ruffling and the extension the ruffles, capable of fully enveloping particles. The extension and rolling over of ruffles (like a wave washing over the particle) was also frequently seen during FcγR-mediated phagocytosis (Fig. 12). In a nice series of experiments involving 2D imaging and scanning electron microscopy, Patel and Harrison (13) proposed a model in which ruffles help phagocytes to capture iC3b-opsonized particles, but, according to this model, ruffles subsequently collapse adjacent to particles and the particles are assumed to slowly sink into the cell. Our data suggest a different model in which CR3 signaling promotes ruffles, as well as ruffle extensions capable of enveloping particles (the ruffles extend and become an enveloping, cup-like structure, analogous to the transition of a curling wave to a tunnel wave). However, we observed that extended ruffles could retract, in accord with Patel and Harrison (13), and reappear, during both FcγR-mediated and CR3-mediated phagocytosis, even when the particles were almost completely enclosed.

Our findings that ITAM adaptors and Syk are prerequisites for CR3-mediated membrane protrusions provide an explanation for the observations by Jaumouillé *et al*. (15) that CR3 can induce phagocytic cups and the Syk inhibitor piceatannol decreases phagocytosis efficiency by ∼50 %. The authors deduced that Syk recruits vinculin to iC3b-opsonized microspheres, which then acts as a clutch to enable force coupling. However, the authors did not identify a mechanism of Syk activation. Moreover, important distinctions compared with FcγRs were also not identified; for example, FcγR-mediated cups can robustly emerge from any point of contact, including the tips or sides of filopodia, whereas CR3-mediated cup formation is inefficient and cups typically emanate from membrane ruffles.

Mocsai *et al*. (25) previously reported that integrins can couple with the ITAM adaptors FcR γ chain and DAP12, but, to date, the roles of these adaptors in phagocytosis mediated by the integrin CR3 have not been determined. We showed that deletion of the ITAM adaptors FcR γ chain and DAP12 phenocopies conditional deletion of Syk, suggesting that CR3-mediated phagocytic ruffles and cups involve CR3 signaling to Syk *via* ITAM adaptor proteins. We infer that the noncovalent interactions between FcγRs and the FcR γ chain are much more effective than equivalent interactions between available ITAM adaptors and CR3. Ultimately, as elegantly demonstrated by Greenberg *et al*. (39), the triggering of phagocytic cups appears to require sufficient clustering of the Syk kinase domain so that tyrosine kinase activity exceeds that of tyrosine phosphatases, such as Shp1 and Shp2 (40), consistent with the notion that phagocytosis is regulated by the balance between inhibitory and activating signaling (20). Indeed, as alluded to in the Results section and shown in Figure 12, the paucity of phagocytic cup formation in *Fcer1g*^−/−^ (FcR γ chain KO) vis-à-vis NOTAM macrophages, presented with dual IgG- and complement C3b/iC3b-opsonized RBCs, can probably be explained by greater inhibitory FcγRIIb activity in *Fcer1g*^−/−^ macrophages (which lack surface expression of FcγRI, FcγRIII, and FcγRIV), unlike NOTAM macrophages (which express the α-chains of FcγRI, FcγRIII, and FcγRIV, each of which can compete with the negative regulator FcγRIIb for opsonic IgG binding).

One of the surprising findings in our study was that FcγR-mediated phagocytic cups become aberrantly elongated and cyclindrical in macrophages lacking CR3, suggesting that FcγRs recruit CR3 for spatiotemporally coordinated cup squeezing and cup closure. Notably, in WT macrophages, the tight closing of cups during FcγR-mediated phagocytosis frequently pinched off the tip of RBCs, possibly serving as an immune defense mechanism to lyse and kill cells (Fig. 12; Videos S2 and S4). CR3 has previously been implicated in FcγR-mediated phagocytosis, for example, anti-CR3 blocking antibodies have been shown to impair the phagocytosis of IgG-opsonized RBCs (41, 42), and neutrophils from patients with leukocyte adhesion deficiency, an autosomal recessive disorder caused by mutations of the β_2_-chain of CR3, exhibit decreased ability to ingest IgG-opsonized RBCs (43). Furthermore, Jongstra-Bilen *et al*. (44) confirmed that blocking of CR3 with specific antibodies impairs FcγR-mediated phagocytosis of IgG-opsonized RBCs, and the authors showed that stimulation of FcγRs both mobilizes and recruits CR3 to the phagocytic cup. Thus, blocking antibody studies, together with our data showing elongated and delayed phagocytic cup closing in CR3-deficient macrophages, strongly suggest that FcγRs recruit CR3 for efficient phagocytic cup formation (Fig. 12).

One of the potential weaknesses of our study was the use of KO mouse models, which are susceptible to genetic compensation (45, 46). We previously encountered genetic robustness when we found that conditional deletion of *Rhoa* (RhoA) in macrophages is compensated by increased protein expression of RhoB and vice versa (47). In the case of experiments with dual IgG-/complement-opsonized particles, it was critical to abolish FcγR-mediated phagocytosis using macrophages from homozygous NOTAM or FcR γ chain KO mice so that the CR-mediated responses could be fully isolated. As proof of concept, we confirmed loss-of-function by showing that the ingestion of singly IgG-opsonized particles is fully blocked in homozygous NOTAM or FcR γ chain KO macrophages. Furthermore, macrophages from homozygous CR3 KO mice did not ingest dual IgM–opsonized/complement-opsonized particles, confirming that CR-mediated phagocytosis was indeed mediated by CR3, without compensation by CRIg, encoded by *Vsig4*, which is also expressed in peritoneal macrophages. Deletion of the β_2_-chain, which is shared by both CR3 and CR4 (expressed in mouse dendritic cells), prevented the possibility of CR4 compensating for loss of CR3. The situation with Syk could have been complicated by compensatory expression of the tyrosine kinase Zap70, which was otherwise not expressed in WT peritoneal macrophages. However, myeloid-restricted deletion of Syk completely blocked ingestion of IgG-opsonized particles by macrophages, indicating that Zap70 does not compensate for loss of Syk. Another limitation of our study was the relatively small number of WT and KO mice used for each group because analyses at this level of replicate hierarchy have low statistical power. Instead, we used individual resident cells (macrophages) as biological replicates (48).

In conclusion, our results, schematically illustrated in Figure 12, indicate that (1) CR3 mediates Syk-independent partial or complete sinking phagocytosis of complement-opsonized particles (2), CR3 induces Syk-dependent ruffling and phagocytic cups *via* the ITAM adaptors FcR γ chain and DAP12, and (3) CR3 contributes to FcγR-mediated phagocytic cup formation and closure.

## Experimental procedures

### Mice

The generation of NOTAM mice, which harbor two inactivation (tyrosine to phenylalanine) mutations in the tyrosine-based activation motif of the Fc receptor γ chain, was previously described by de Haij *et al* (21). Mice lacking the Fc receptor γ chain, encoded by *Fcer1g*, were generated by Takai *et al* (49) and DAP12-deficient (*Tyrobp*^−/−^) mice were produced by Bakker *et al* (50). These mice were crossed to derive *Fcer1g*/*Tyrobp* dKO mice (25). Myeloid-restricted *Syk* cKO mice were produced by crossing *Syk*^fl^ (B6.129P2-*Syk*^tm1.2Tara^/J) mice (51), in which exon 1 has been floxed (flanked by *loxP* sites), with LysM-Cre (B6.129P2-*Lyz2*^tm1(cre)Ifo^/J) mice (52). LysM-Cre knock-in mice were produced by introducing DNA coding for Cre recombinase into the start of the gene *Lyz2*, encoding lysozyme 2, which results in selective expression of Cre recombinase in myeloid cells, including granulocytes and macrophages. Mice lacking *Itgb2* (integrin beta 2), which codes for β_2_ integrin, also known as CD18, have been previously described (53). Homozygous complement C5 null (B10.D2-Hc^0^ H2^d^ H2-T18^c^/oSnJ) mice were obtained from The Jackson Laboratory. All work with animals was performed in accordance with the German Animal Welfare Act (Tierschutzgesetz) and Directive 2010/63/EU (European Parliament and Council of the European Union, 2010) and approved by the local ethics committee of the University of Münster.

### Isolation of mouse resident peritoneal macrophages

Methods for the isolation and handling of mouse resident peritoneal macrophages have been previously described (31, 54). In brief, mice were sacrificed by an overdose of isoflurane followed by cervical dislocation, and the peritoneal cavity was lavaged (2 × 4.5 ml) *via* a 24-G plastic catheter (B. Braun) using ice-cold Hank’s balanced salt solution without Ca^2+^ or Mg^2+^ (14175-046; Gibco, Life Technologies) (55). After centrifugation (300*g* for 6.5 min), cells were resuspended in bicarbonate-free RPMI 1640 medium containing 20-mM Hepes (R7388-500ML; Sigma-Aldrich) and supplemented with 10% heat-inactivated fetal calf serum (FCS), 100 U/ml penicillin, and 100 μg/ml streptomycin (pH 7.4). The cells were seeded into fibronectin-coated μ-slide I chambers (Ibidi), which each consist of a 500 mm × 5 mm × 0.4 mm channel flanked by 2-ml reservoirs (55), and placed in a humidified incubator (37 °C). After 2-h incubation, μ-slide I chambers were washed once and then each chamber was filled with 1-ml RPMI 1640 medium containing sodium bicarbonate (R8758-500ML; Sigma-Aldrich), as well as 10% FCS and antibiotics and incubated overnight in a humidified incubator with 5% CO_2_ at 37 °C. After overnight incubation, the medium was switched to bicarbonate-free RPMI 1640 medium containing 20-mM Hepes, 1-mM N-(2-mercaptopropionyl)glycine (an antioxidant), 10% heat-inactivated FCS, and antibiotics. The μ-slide I chambers were then placed on an anodized aluminum rack and transferred to a CO_2_-free incubator maintained at 37 °C.

### RNA sequence analyses

Mouse resident peritoneal cells were labeled with Alexa Fluor 488–conjugated anti-F4/80 antibodies, washed, and resuspended in autoMACS running buffer (Miltenyi Biotec), which contains PBS, 2-mM EDTA, 0.5% bovine serum albumin, and 0.09% sodium azide (pH 7.2). Purification of F4/80^+^ cells (mouse macrophages) was performed using a BD FACSAria II (or FACSAria III) cell sorter (BD Biosciences). The recovered suspension of F4/80^+^ cells was centrifuged at 300*g* for 5 min, and the supernatant was removed, followed by isolation of total RNA by solid-phase extraction using a Direct-zol RNA MicroPrep kit (Zymo Research), according to the manufacturer’s instructions. The pellet of F4/80^+^ cells was lysed with 300-μl TRIzol (Thermo Fisher Scientific). Next, 300-μl analytical (100%) ethanol was added and the mixture was transferred to a Zymo-Spin IC column, inserted into a collection tube. After wash steps, as well as DNase treatment for 15 min, involving several centrifugations at 12,000*g*, purified total RNA was captured in a silica column. Using RNase-free water, concentrated RNA was eluted from the silica column and collected in a DNase-/RNase-free safe-lock tube. Isolated RNA samples were tested for integrity using RNA ScreenTape (Agilent Technologies) and stored at −80 °C.

Next-generation sequencing was performed using a NextSeq 500 Sequencing System (Illumina). Samples were prepared using a TruSeq RNA sample preparation kit (Illumina), which involved the following steps: purification and fragmentation of mRNA, first- and second-strand cDNA synthesis, end repair, adenylation of 3′ ends, ligation with adaptors, and PCR amplication. RNA-Seq data were analyzed using the Tuxedo suite, an open access set of applications for ultrafast alignment of short reads to the genome, recognition of splice junctions, and differential expression analysis.

### Isolation of hRBCs

Using a 21-G butterfly needle (Venofix A, B. Braun), 1- to 2-ml peripheral venous blood was collected from a healthy donor into a polypropylene tube (S-Monovette 7.5-ml LH, Sarstedt) containing 120 IU lithium heparin. The blood was transferred *via* a 70- μm cell strainer (542070, EASYstrainer, Greiner Bio-One) into a 50-ml polypropylene tube (Sarstedt), and then 1 ml of the filtered blood was pipetted into a round bottom 2.0 ml (polypropylene) microcentrifuge tube and centrifuged at 300*g* for 5 min at 18 °C. The supernatant, plasma, and buffy coat were removed, and 100 μl of the red blood cells was transferred into a round bottom 2.0-ml tube and diluted 1:1 with RPMI 1640-Hepes medium containing 10% heat-inactivated FCS and antibiotics. The 1:1 diluted hRBCs were placed on ice.

### Antibody opsonization and complement-induced hemolysis assays

Activation of the terminal pathway of the complement cascade was indirectly measured by imaging lysis of red blood cells, indexed as loss of cytosolic green fluorescent calcein or red fluorescent pHrodo Red. First, 8 μl from a 1:1 diluted red blood cell suspension was added to 992-μl RPMI 1640–Hepes medium containing 10% heat-inactivated FCS and antibiotics. Second, 400 μl of the 8:1000 diluted suspension was pipetted into a 2.0 ml microcentrifuge tube and centrifuged at 300*g* for 5 min at 18 °C. Third, the supernatant was discarded and the cells were resuspended in 400-μl modified RPMI 1640–Hepes medium supplemented with 10-μM calcein/AM (ester of calcein), diluted from a freshly prepared 10-mM stock solution of calcein/AM (dissolved in dimethylsulfoxide with 20% Pluronic F-127) and incubated at 37 °C. After 5 min, 0.4-μl CMO (C10045; Thermo Fisher Scientific), a red fluorescent plasma membrane marker, was added, and the mixture was incubated for a further 5 min, followed by two wash steps, and the pellet of dual fluorescently labeled red blood cells was resuspended in 400-μl modified RPMI 1640–Hepes medium. Alternatively, hRBCs were loaded with pHrodo Red using its AM form. In brief, 1-μl pHrodo Red/AM (P35372, Thermo Fisher Scientific) was added to 10-μl PowerLoad (provided with P35372, Thermo Fisher Scientific) and then 10 μl of this mixture was added to 1 ml of hRBCs, which had been consecutively diluted 1:1 and then 8:1000 in the modified RPMI 1640–Hepes medium. The cells were loaded with pHrodo Red for 15 min at RT, followed by two wash steps.

Opsonization of hRBCs with mouse IgG antibody was achieved by incubating 200 μl of the suspension with 2-μl mouse anti-CD235a monoclonal (clone HIR2) anti-human CD235a (IgG2b) antibody, 1 mg/ml (MA1-20893; Thermo Fisher Scientific) aliquots of which were stored at −20 °C. Notably, CD235a (also known as glycophorin A) is a human erythrocyte–specific membrane sialoglycoprotein. After 8-min incubation at 37 °C with intermittent pipetting to reduce hemagglutination, 50 μl of the CMO and calcein stained, and IgG-opsonized, hRBCs were rapidly mixed with 50-μl WT mouse serum, prepared in advance from coagulated mouse whole blood, and a timer was started. The mixture was quickly pipetted into a μ-slide I chamber and imaged by time-lapse spinning disk confocal microscopy. Alternatively, hRBCs were opsonized with mouse monoclonal (clone 88-O) anti-blood group A, B, H antigens (IgM) antibody (1 mg/ml aliquots of which were stored at −20 °C), which were generated using H-antigen of hRBCs (DBGA-0197; Creative Diagnostics). hRBCs were initially incubated with IgM at RT, rather than at 37 °C, and time-lapse spinning disk confocal microscopy was performed after mixing 50 μl of the IgM/hRBCs mixture with 50-μl WT mouse serum. The mixture slowly warmed, over 6 min, on the stage of the microscope to a maximum temperature of ∼33 °C. Images at a single focal plane were captured at a rate of 1 time point every 5 s and analyzed using the open-source software ImageJ (56).

### Calibration of pHrodo red-labeled hRBCs

The fluorescence intensity of pHrodo Red increases with decreasing pH, as occurs during phagosome maturation. A plot of intracellular pHrodo Red fluorescence intensity as a function of extracellular pH was produced using an intracellular pH calibration buffer kit (P35379; Thermo Fisher Scientific). hRBCs were loaded with pHrodo Red as described above, except the cells were suspended in PBS instead of in the RPMI 1640 medium. In contrast to the instructions provided with the calibration kit, the ionophores valinomycin and nigericin were not added to the hRBC suspension. pHrodo Red–loaded hRBCs were centrifuged and resuspended in the buffer with a pH of 7.5, 6.5, 5.5, or 4.5. Z-stacks (22 slices with 0.8-μm step size) were obtained *via* a 60×/1.49 oil immersion objective lens using a 561-nm laser at a rate of 15 s per time point.

### 3D time-lapse imaging of phagocytosis

Cells were imaged *via* the Apochromat TIRF 60×/1.49 oil immersion objective lens of a Nikon Eclipse Ti microscope. The inverted microscope was connected to a spinning disk confocal system (UltraVIEW Vox 3D live cell imaging system), which included a Yokogawa CSU-X1 spinning disk scanner, a Hamamatsu C9100-50 EM-CCD camera (1000 × 1000 pixels), and Volocity software. Calcein and Alexa Fluor 488 were excited with a 488-nm laser, whereas CMO and pHrodo Red were excited *via* a 561-nm laser. Typically, Z-stacks (22 slices with 0.8-μm step size) were captured for each channel using 2 × 2 binning (giving 500 × 500 pixels per image) at a rate of 15 s per time point. Focus drift was prevented using the Nikon Perfect Focus System, which maintains the position of the coverslip in the z-axis by reflecting near-infrared light (870 nm) and detecting it with a charge-coupled device line sensor. The temperature was maintained at 37 °C using an Okolab all-in-one stage incubator (Okolab) and an objective lens heating mantle (Scientific Instruments).

Macrophages were labeled fluorescently green using Alexa Fluor 488–conjugated rat anti-mouse F4/80 (IgG2a) monoclonal (clone BM8) antibodies (MF48020, Thermo Fisher Scientific). The antibody (0.2 mg/ml) was diluted 1:40, and 100 μl was applied to cells, seeded overnight in the 100-μl channel of a fibronectin-coated μ-slide I chamber, for 20 min at 37 °C, followed by a wash step. During this incubation time, a 400-μl aliquot of hRBCs (sequentially diluted 1:1 and 4:2000, and prewarmed to 37 °C) was incubated with CMO (diluted 1:1000) for 5 min, followed by two wash steps. Next, the CMO-labeled cells were incubated for 8 min with mouse monoclonal (clone HIR2) anti-human CD235a (IgG2b) antibody, diluted 1:400, and intermittently pipetted up and down to prevent hemagglutination. Alternatively, cells were dual opsonized with IgG and C3b/iC3b by incubating 2× concentrated hRBCs, labeled with CMO, with mouse anti-CD235a monoclonal (clone HIR2) anti-human CD235a (IgG2b) antibody (diluted 1:200) for 4 min, followed by 1:1 mixing with C5 null mouse serum and a further 4-min incubation period with intermittent up and down pipetting. The same approach was used for dual opsonization with IgM and C3b/iC3b, except the hRBCs were preloaded with pHrodo Red (instead of stained with CMO), and the cell suspension of 2× concentrated hRBCs and anti-blood group A, B, H antigens (IgM) and antibody (diluted 1:200) was immediately mixed 1:1 with complement C5 null mouse serum at RT (required for efficient IgM binding). Note that IgG or IgM opsonization activates the classical complement cascade, leading to the deposition of C3b, which is cleaved to iC3b by complement factor I. A 100-μl suspension of red fluorescent and IgG-opsonized, dual IgG–opsonized /iC3b-opsonized, or dual IgM–opsonized/iC3b-opsonized hRBCs was presented to fluorescent green antibody–labeled macrophages and time-lapse Z-stacks were obtained by spinning disk confocal microscopy. Phagocytosis assays were performed using the RPMI 1640 medium containing 20-mM Hepes, 1-mM N-(2-mercaptopropionyl)glycine, 10% heat-inactivated FCS, and antibiotics.

### Statistics

A nonparametric Mann–Whitney U test or Kruskal–Wallis one-way ANOVA on ranks was used to test for statistical differences using α = 0.05. Post hoc multiple comparisons were made using Dunn’s test. Statistical analyses were performed using OriginPro 2020 (OriginLab) or earlier versions. Data are shown as box plots, including the 25th percentile, median, 75th percentile, and outliers.

## Data availability

All data are contained within the manuscript.

## Conflict of interest

The authors declare that they have no conflicts of interest with the contents of this article.
