# Supplementary material for: Complement receptor 3 mediates both sinking phagocytosis and phagocytic cup formation via distinct mechanisms
Source: J Biol Chem. 2021 Jan 8;296:100256. doi: 10.1016/j.jbc.2021.100256 (PMC7948798; doi:10.1016/j.jbc.2021.100256)
Supplement: Figures S1 and S2 [file mmc1.pdf]

Supporting Information

Supplemental figures

Figure S1. RNA-sequence analysis and real-time hemolysis assay for IgG-opsonized human red blood cells. *A*, levels of mRNA expression for genes encoding Fcγ receptors (FcγRs) and upstream signaling proteins. RNA was isolated from purified resident mouse peritoneal F4/80$^+$ cells (macrophages). Data are mean ± s.d. (n = 3 independent experiments). *B*, schematic diagram of FcγR-mediated signaling. FcγRI, FcγRIII and FcγRIV each associate with an Fc receptor γ-chain (homodimeric adaptor protein) containing an immunoreceptor tyrosine-based activation motif (ITAM), whereas the cytosolic tail of FcγRIIb contains an immunoreceptor tyrosine-based inhibition motif (ITIM). Dual phosphorylation of an ITAM generates specific docking sites for the binding and activation of the tyrosine kinase Syk, also known as spleen (S) tyrosine (denoted by the one-letter symbol Y) kinase (k), whereas phosphorylation of ITIM promotes the binding and activation of the tyrosine phosphatases Shp1 and Shp2. *C*, schematic diagram showing how IgG-opsonized human red blood cells (hRBCs) trigger the activation of the classical complement cascade, leading to the deposition of complement C3b and the formation of the membrane attack complex (MAC), which can induce hemolysis. *D*, real-time imaging of hemolysis by spinning disk confocal microscopy. The cytosol of hRBCs was loaded with the green fluorescent probe Calcein and the plasma membrane was labeled red fluorescent with CellMask Orange. Unopsonized or IgG-opsonized hRBCs were incubated with wild-type (WT) mouse serum as indicated. Scale bars, 10 µm. *E*, loss of Calcein fluorescence was used as an index of hemolysis. *F*, representative cumulative hemolysis plot for unopsonized and IgG-opsonized hRBCs incubated with WT mouse serum.

Figure S2. Complement receptor expression and real-time hemolysis assay for IgM-opsonized human red blood cells. *A*, schematic diagram showing the mouse complement receptors which bind to complement C3b, a proteolytic fragment of complement C3, and complement iC3b, produced by cleavage of complement C3b by complement factor I. *B*, levels of mRNA expression for genes encoding complement receptors (CRs). RNA was isolated from purified resident mouse peritoneal F4/80$^+$ cells (macrophages). Data are mean ± s.d. (n = 3 independent experiments). Note that CR3 is an integrin, a heterodimeric receptor which mediates cell-extracellular matrix adhesion, consisting of the transmembrane proteins CD18, the β subunit (β_2; encoded by *Itgb2*), and CD11b (encoded by *Itgam*), the α subunit. *C*, schematic diagram showing how IgM-opsonized human red blood cells (hRBCs) trigger the activation of the classical complement cascade, leading to deposition of complement C3b and formation of the membrane attack complex (MAC), which can induce hemolysis. *D*, fluorescence intensity of pHrodo Red-loaded human red blood cells (hRBCs) incubated in media with varying pH values. The pH-sensitive, fluorescent probe was loaded into hRBCs using its acetoxymethyl ester derivative. *E*, real-time imaging of hemolysis by spinning disk confocal microscopy. The cytosol of hRBCs was loaded with pHrodo Red and unopsonized or IgG-opsonized hRBCs were incubated with wild-type (WT) mouse serum as indicated. Scale bars, 10 µm. *F*, loss of cytosolic pHrodo Red fluorescence was used as an index of hemolysis. *G*, representative cumulative hemolysis plot for unopsonized and IgM-opsonized hRBCs incubated with WT mouse serum. Importantly, hRBCs were incubated with IgM antibodies at room temperature before introducing WT serum. The mixture was slowly warmed to ~30 °C on the stage of the microscope during recordings, as indicated above the cumulative plots.

Supplemental videos

Video S1. Ingestion of a IgG-opsonized human red blood cell by a wild-type mouse peritoneal macrophage via phagocytic cup formation. Time-lapse 3D reconstructions of a mouse macrophage (green) and a IgG-opsonized human red blood cell (red) were generated from 17 dual-channel z-stacks captured by spinning disk confocal imaging at a rate of 1 timepoint every 15 s. The macrophage was labeled green fluorescent with Alexa Fluor 488-conjugated anti-F4/80 antibodies and the membrane of the red blood cell was labeled red fluorescent with the plasma membrane stain CellMask Orange. The grid spacings represent 8.93 μm.

Video S2. Ingestion of an IgG-opsonized human red blood cell by an unpolarized (spherical) wild-type mouse peritoneal macrophage via phagocytic cup formation. Time-lapse extended focus images (30 μm × 30 μm) of a spherical mouse macrophage (green) and a IgG-opsonized human red blood cell (red) were generated from 38 dual-channel z-stacks captured by spinning disk confocal imaging at a rate of 1 timepoint every 15 s. The macrophage was labeled green fluorescent with Alexa Fluor 488-conjugated anti-F4/80 antibodies and the membrane of the red blood cell was labeled red fluorescent with the plasma membrane stain CellMask Orange.

Video S3. Ingestion of a dual IgM- and complement C3b/iC3b-opsonized human red blood cell by a wild-type mouse peritoneal macrophage via phagocytic cup formation. Time-lapse 3D reconstructions of a mouse macrophage (green) and a dual opsonized human red blood cell (red) were generated from 23 dual-channel z-stacks captured by spinning disk confocal imaging at a rate of 1 timepoint every 15 s. The macrophage was labeled green fluorescent with Alexa Fluor 488-conjugated anti-F4/80 antibodies and the cytosol of the red blood cell was loaded with the pH-sensitive, red fluorescent probe pHrodo Red. The grid spacings represent 6.83 μm.

Video S4. Ingestion of an IgG-opsonized human red blood cell by a wild-type mouse peritoneal macrophage via squeezing and phagocytic cup formation. Time-lapse extended focus images (47.3 μm (width) × 48.5 μm) of a mouse macrophage (green) and an IgG-opsonized human red blood cell (red) were generated from 16 dual-channel z-stacks captured by spinning disk confocal imaging at a rate of 1 timepoint every 15 s. The macrophage was labeled green fluorescent with Alexa Fluor 488-conjugated anti-F4/80 antibodies and the membrane of the red blood cell was labeled red fluorescent with the plasma membrane stain CellMask Orange.

Video S5. Ingestion of a IgG-opsonized human red blood cell by a wild-type mouse peritoneal macrophage via squeezing and phagocytic cup formation in 3D. Time-lapse 3D reconstructions of mouse macrophages (green) and IgG-opsonized human red blood cells (red) were generated from 8 dual-channel z-stacks captured by spinning disk confocal imaging at a rate of 1 timepoint every 15 s. The macrophages were labeled green fluorescent with Alexa Fluor 488-conjugated anti-F4/80 antibodies and the membranes of the red blood cells were labeled red fluorescent with the plasma membrane stain CellMask Orange. The grid spacings represent 10.32 μm.

Video S6. Consecutive ingestion of two IgG-opsonized human red blood cells by a wild-type mouse peritoneal macrophage via phagocytic cup formation. Time-lapse 3D reconstructions of a mouse macrophages (green) and IgG-opsonized human red blood cells

(red) were generated from 16 dual-channel z-stacks captured by spinning disk confocal imaging at a rate of 1 timepoint every 15 s. The macrophages were labeled green fluorescent with Alexa Fluor 488-conjugated anti-F4/80 antibodies and the membranes of the red blood cells were labeled red fluorescent with the plasma membrane stain CellMask Orange. The grid spacings represent 10.32 μm.

Video S7. Lack of ingestion of IgG-opsonized human red blood cells by macrophages from a conditional *Syk* knockout (*Syk* cKO) mouse. Time-lapse 3D reconstructions of *Syk* cKO macrophages (green) and IgG-opsonized human red blood cells (red) were generated from 40 dual-channel z-stacks captured by spinning disk confocal imaging at a rate of 1 timepoint every 15 s. The macrophages were labeled green fluorescent with Alexa Fluor 488-conjugated anti-F4/80 antibodies and the membranes of the red blood cells were labeled red fluorescent with the plasma membrane stain CellMask Orange. The grid spacings represent 8.39 μm.

Video S8. Ingestion of dual IgM- and complement C3b/iC3b-opsonized human red blood cells by *Syk* conditional knockout (*Syk* cKO) macrophages by sinking phagocytosis. The 48.8 μm × 48.8 μm optical sections (x-y plane) of *Syk* cKO macrophages (green) and dual IgM- and complement C3b/iC3b-opsonized human red blood cells (red) were obtained from 116 dual-channel z-stacks captured by spinning disk confocal imaging at a rate of 1 timepoint every 15 s. The macrophages were labeled green fluorescent with Alexa Fluor 488-conjugated anti-F4/80 antibodies and the cytosol of the red blood cells were loaded with the pH-sensitive, red fluorescent probe pHrodo Red.

Video S9. Ingestion of a dual IgM- and complement C3b/iC3b-opsonized human red blood cell by a *Syk* conditional knockout (*Syk* cKO) macrophage by sinking phagocytosis. The 30.5 μm × 30.5 μm optical sections (x-y plane) of a *Syk* cKO macrophage (green) and dual IgM- and complement C3b/iC3b-opsonized human red blood cells (red) were obtained from 113 dual-channel z-stacks captured by spinning disk confocal imaging at a rate of 1 timepoint every 15 s. The macrophages were labeled green fluorescent with Alexa Fluor 488-conjugated anti-F4/80 antibodies and the cytosol of the red blood cells were loaded with the pH-sensitive, red fluorescent probe pHrodo Red.

Video S10. Ingestion of a dual IgM- and complement C3b/iC3b-opsonized human red blood cell by a *Fcer1g*/*Tyrobp* (FcR γ-chain/DAP12) double knockout (dKO) macrophage by a combination of sinking phagocytosis and membrane protrusive activity. Time-lapse 3D reconstructions of the *Fcer1g*/*Tyrobp* dKO macrophage (green) and dual IgM- and complement C3b/iC3b-opsonized human red blood cells (red) were generated from 22 dual-channel z-stacks captured by spinning disk confocal imaging at a rate of 1 timepoint every 15 s. The macrophages were labeled green fluorescent with Alexa Fluor 488-conjugated anti-F4/80 antibodies and the cytosol of the red blood cells were loaded with the pH-sensitive, red fluorescent probe pHrodo Red. The grid spacings represent 2.56 μm.

Video S11. Ingestion of a dual IgM- and complement C3b/iC3b-opsonized human red blood cell by a *Fcer1g*/*Tyrobp* (FcR γ-chain/DAP12) double knockout (dKO) macrophage by slow sinking phagocytosis. The 26.3 μm × 26.3 μm optical sections (x-y plane) of a *Fcer1g*/*Tyrobp* dKO macrophage (green) and dual IgM- and complement C3b/iC3b-opsonized human red blood cells (red) were obtained from 60 dual-channel z-stacks captured by spinning disk confocal imaging at a rate of 1 timepoint every 15 s. The macrophages were labeled green fluorescent with Alexa Fluor 488-conjugated anti-F4/80 antibodies and the cytosol of the red blood cells were loaded with the pH-sensitive, red fluorescent probe pHrodo Red.

Video S12. Ingestion of a dual IgM- and complement C3b/iC3b-opsonized human red blood cell by a *Fcer1g/Tyrobp* (FcR γ-chain/DAP12) double knockout (dKO) macrophage by moderately slow sinking phagocytosis. The 25.1 μm × 25.1 μm optical sections (x-y plane) of a *Fcer1g/Tyrobp* dKO macrophage (green) and dual IgM- and complement C3b/iC3b-opsonized human red blood cells (red) were obtained from 60 dual-channel z-stacks captured by spinning disk confocal imaging at a rate of 1 timepoint every 15 s. The macrophages were labeled green fluorescent with Alexa Fluor 488-conjugated anti-F4/80 antibodies and the cytosol of the red blood cells were loaded with the pH-sensitive, red fluorescent probe pHrodo Red.

Video S13. Ingestion of two dual IgM- and complement C3b/iC3b-opsonized human red blood cells by a *Tyrobp* (DAP12) knockout (KO) macrophage by slow sinking phagocytosis with varying degrees of membrane protrusive activity. The 25.1 μm × 25.1 μm optical sections (x-y plane) of a *Tyrobp* KO macrophage (green) and dual IgM- and complement C3b/iC3b-opsonized human red blood cells (red) were obtained from 65 dual-channel z-stacks captured by spinning disk confocal imaging at a rate of 1 timepoint every 15 s. The macrophages were labeled green fluorescent with Alexa Fluor 488-conjugated anti-F4/80 antibodies and the cytosol of the red blood cells were loaded with the pH-sensitive, red fluorescent probe pHrodo Red.

Video S14. Complete or partial ingestion of several dual IgM- and complement C3b/iC3b-opsonized human red blood cells by a *Tyrobp* (DAP12) knockout (KO) macrophage by sinking phagocytosis with varying degrees of membrane protrusive activity. The 25.1 μm × 25.1 μm optical sections (x-y plane) of a *Tyrobp* KO macrophage (green) and dual IgM- and complement C3b/iC3b-opsonized human red blood cells (red) were obtained from 98 dual-channel z-stacks captured by spinning disk confocal imaging at a rate of 1 timepoint every 15 s. The macrophages were labeled green fluorescent with Alexa Fluor 488-conjugated anti-F4/80 antibodies and the cytosol of the red blood cells were loaded with the pH-sensitive, red fluorescent probe pHrodo Red.

Video S15. Macrophages lacking complement receptor 3 (*Itgb2* knockout (KO) macrophages) exhibit elongated, cylindrical phagocytic cups during Fcγ receptor-mediated phagocytosis. Time-lapse 3D reconstructions of the *Itgb2* KO macrophages (green) were generated from 40 dual-channel z-stacks captured by spinning disk confocal imaging at a rate of 1 timepoint every 15 s. The macrophages were labeled green fluorescent with Alexa Fluor 488-conjugated anti-F4/80 antibodies. The grid spacings represent 7.63 μm.

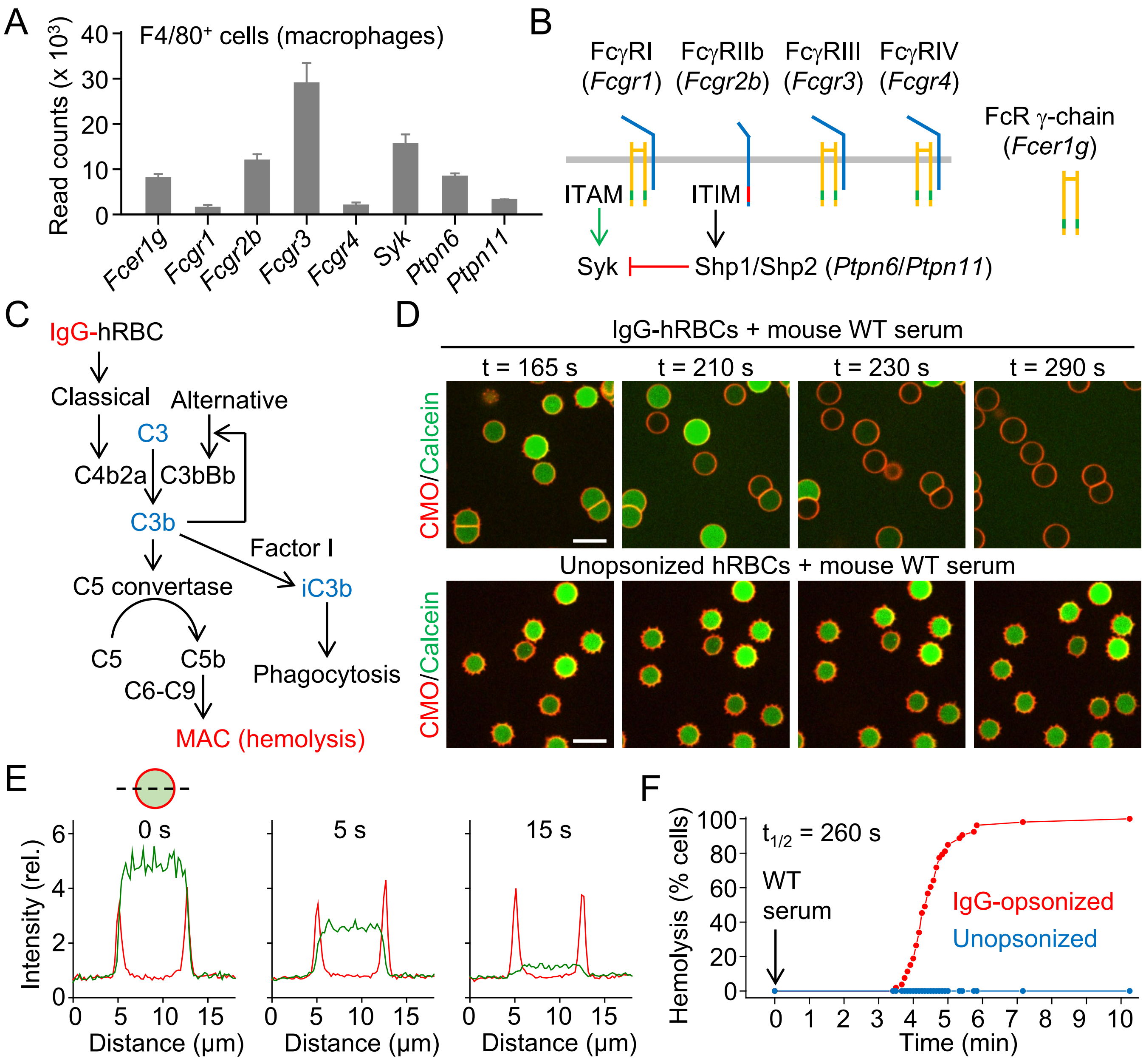
A
F4/80+ cells (macrophages)
Read counts (x 10^3)
Fcer1g
Fcgr1
Fcgr2b
Fcgr3
Fcgr4
Syk
Ptpn6
Ptpn11
B
FcγRI (Fcgr1)
FcγRIIb (Fcgr2b)
FcγRIII (Fcgr3)
FcγRIV (Fcgr4)
FcR γ-chain (Fcer1g)
ITAM
ITIM
Syk
Shp1/Shp2 (Ptpn6/Ptpn11)
C
IgG-hRBC
Classical
Alternative
C3
C4b2a
C3bBb
C3b
Factor I
iC3b
C5 convertase
C5
C5b
C6-C9
Phagocytosis
MAC (hemolysis)
D
IgG-hRBCs + mouse WT serum
t = 165 s
t = 210 s
t = 230 s
t = 290 s
CMO/Calcein
Unopsonized hRBCs + mouse WT serum
CMO/Calcein
E
0 s
5 s
15 s
Intensity (rel.)
Distance (µm)
Distance (µm)
Distance (µm)
F
t1/2 = 260 s
WT serum
IgG-opsonized
Unopsonized
Hemolysis (% cells)
Time (min)

Figure S1

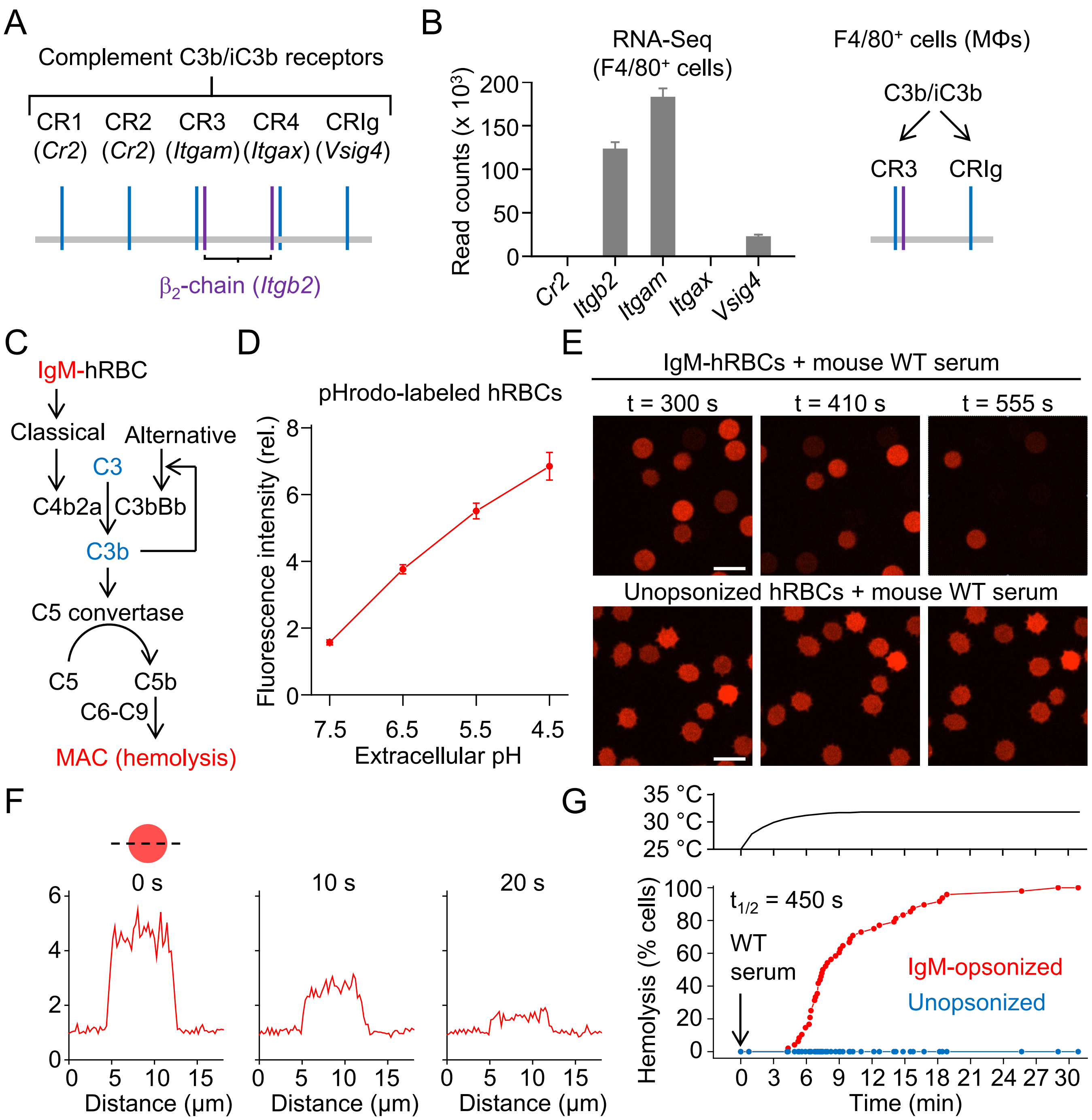
A
Complement C3b/iC3b receptors
CR1 (Cr2)
CR2 (Cr2)
CR3 (Itgam)
CR4 (Itgax)
CRIg (Vsig4)
β2-chain (Itgb2)
B
RNA-Seq (F4/80+ cells)
Read counts (x 10^3)
0
50
100
150
200
Cr2
Itgb2
Itgam
Itgax
Vsig4
F4/80+ cells (MΦs)
C3b/iC3b
CR3
CRIg
C
IgM-hRBC
Classical
Alternative
C3
C4b2a
C3bBb
C3b
C5 convertase
C5
C5b
C6-C9
MAC (hemolysis)
D
pHrodo-labeled hRBCs
Fluorescence intensity (rel.)
0
2
4
6
8
7.5
6.5
5.5
4.5
Extracellular pH
E
IgM-hRBCs + mouse WT serum
t = 300 s
t = 410 s
t = 555 s
Unopsonized hRBCs + mouse WT serum
F
0 s
10 s
20 s
0
2
4
6
0 5 10 15
Distance (μm)
G
35 °C
30 °C
25 °C
Hemolysis (% cells)
0
20
40
60
80
100
t1/2 = 450 s
WT serum
IgM-opsonized
Unopsonized
0 3 6 9 12 15 18 21 24 27 30
Time (min)

Figure S2
